# Characterization of choroid plexus in the preterm rabbit pup following subcutaneous administration of recombinant human IGF-1/IGFBP-3

**DOI:** 10.1186/s12987-023-00460-1

**Published:** 2023-08-15

**Authors:** Niklas Ortenlöf, Suvi Vallius, Helena Karlsson, Claes Ekström, Amanda Kristiansson, Bo Holmqvist, Olga Göransson, Magdaléna Vaváková, Martin Rydén, Galen Carey, Norman Barton, David Ley, Magnus Gram

**Affiliations:** 1 Pediatrics, Department of Clinical Sciences Lund, Lund University, Lund, Sweden; 2 Pediatrics, Department of Clinical Sciences Lund, Lund University, Skåne University Hospital, Lund, Sweden; 3 Imagene-iT AB, Lund, Sweden; 4 Department of Experimental Medical Science, Lund University, Lund, Sweden; 5 Orthopaedics, Department of Clinical Sciences Lund, Lund University, Lund, Sweden; 6 Takeda, Cambridge, MA USA; 7 Oak Hill Bio, Scientific Advisory Board, Boston, MA USA

**Keywords:** Insulin-like growth factor-1, Choroid plexus, Blood-cerebrospinal fluid barrier, Preterm infant, Immature brain, Preterm rabbit pup, Ventricles, Cerebrospinal fluid, Serum, Insulin-like growth factor-1 receptor

## Abstract

Insulin-like growth factor-1 (IGF-1) is essential for normal brain development and regulates essential processes of vascular maturation and stabilization. Importantly, preterm birth is associated with reduced serum levels of IGF-1 as compared to *in utero* levels. Using a preterm rabbit pup model, we investigated the uptake of systemic recombinant human (rh) IGF-1 in complex with its main binding protein IGF-binding protein 3 (BP-3) to the brain parenchyma via the choroid plexus. Five hours after subcutaneous administration, labeled rhIGF-1/rhIGFBP-3 displayed a widespread presence in the choroid plexus of the lateral and third ventricle, however, to a less degree in the fourth, as well as in the perivascular and subarachnoid space. We found a time-dependent uptake of IGF-1 in cerebrospinal fluid, decreasing with postnatal age, and a translocation of IGF-1 through the choroid plexus. The impact of systemic rhIGF-1/rhIGFBP-3 on IGF-1 receptor activation in the choroid plexus decreased with postnatal age, correlating with IGF-1 uptake in cerebrospinal fluid. In addition, choroid plexus gene expression was observed to increase with postnatal age. Moreover, using choroid plexus in vitro cell cultures, gene expression and protein synthesis were further investigated upon rhIGF-1/rhIGFBP-3 stimulation as compared to rhIGF-1 alone, and found not to be differently altered. Here, we characterize the uptake of systemic rhIGF-1/rhIGFBP-3 to the preterm brain, and show that the interaction between systemic rhIGF-1/rhIGFBP-3 and choroid plexus varies over time.

**Supplementary Information:**

The online version contains supplementary material available at 10.1186/s12987-023-00460-1.

## Introduction

The mitogenic peptide insulin-like growth factor 1 (IGF) is involved in the regulation of cell metabolism and angiogenesis, and is an essential element in fetal brain development [1, 2]. Serum levels of IGF-1 are considerably lower following preterm birth compared to the fetus of corresponding gestational age remaining *in utero* [3–5]. Reduced postnatal serum levels of IGF-1 are associated with elevated risk for neurodevelopment impairment, along with poor weight gain and lower brain volumes [5]. Supplementing extremely preterm infants, i.e. born below gestational week 28, with recombinant human (rh) IGF-1 conjugated with rhIGF binding protein-3 (BP-3), aiming to achieve circulatory concentrations of the healthy fetus within normal intrauterine range, resulted in a trend towards decreased occurrence of severe intraventricular hemorrhage (IVH) [6]. Although findings from a range of clinical observational studies [7] have suggested that supplementary treatment with IGF-1 may potentially reduce brain morbidities, including IVH, the molecular mechanisms involved in the IGF-1 mediated protective effects on the immature brain remains to be fully elucidated.

In contrast to insulin, which is suggested to penetrate the blood brain barrier, entry of systemic IGF-1 to the brain has been proposed to take place at the blood–cerebrospinal fluid (CSF) barrier in the choroid plexus (ChP) [2, 8]. The ChP is strategically located in each ventricle of the brain, regulating the production of the CSF, which in turn bathes the central nervous system (CNS) with nutrients and blood-borne signaling molecules and provides adequate shock absorption. The selective transcytosis of systemic IGF-1 through the ChP is believed to involve the IGF-1 receptor (IGF-1R) which is abundantly expressed in the ChP [9]. However, in the immature brain of the preterm rabbit pup, the IGF-1R has been shown to be mainly located at the epithelial border of the ChP, thereby facing the CSF, and to a lesser extent at the endothelial side [3]. Therefore, it is reasonable to consider other putative mechanism of IGF-1 transcytosis through the ChP besides the involvement of the IGF-1R, such as in extracellular vesicles encapsulation. In addition, the IGF-1R is abundantly distributed throughout the preterm rabbit brain, 4 h after postnatal birth. However, expect for ChP, the presence of the IGF-1R was significantly reduced at 96 h postnatal age [3]. Therefore, we aimed to investigate if the uptake of IGF-1 from the circulation to the CSF follows the same temporal pattern as the IGF-1R distribution in the preterm rabbit brain.

Here, we have used a preterm rabbit pup model, where pups are delivered using cesarean section (c.s.) at post-conceptional day 29 (term 31–32 days), to characterize the interaction between systemic rhIGF-1/rhIGFBP-3 and ChP, as well as the uptake of systemic IGF-1 to the brain following subcutaneous (s.c.) administration. The rabbit has been widely used in preclinical studies simulating IVH, cerebral palsy and hypoxia-ischemia [10], and the preterm rabbit pup incorporates many important physiological aspects of the human preterm infant, including reduced IGF-1 levels, risk of developing necrotizing enterocolitis-like disease, and displaying renal immaturity [10, 11]. In addition, the rabbit can contribute to bridge the translational gap between humans and rodents, as the rabbit brain develops in the perinatal period with a temporal white matter development corresponding to that of the human [10], as well as having a more complex brain structure compared to rodents [12].

In the present study, the effects of IGF-1 on the ChP of the immature brain was investigated following s.c. administration of rhIGF-1/rhIGFBP-3. We observed that postnatal age at time-point of administration affects concentrations of IGF-1 in the circulation and the CSF, and the IGF-1R activation in the ChP. Finally, studies in vitro show that the rhIGF-1/rhIGFBP-3 complex and non-complexed rhIGF-1 exhibit similar effects on the ChP epithelium.

## Methods

### Animals

The animal protocols were approved by the Swedish Animal Ethics Committee in Lund (Dnr: 5.8.18–06020/2019 and 5.8.18–11,990/2021). We used the well-established preterm rabbit pup model in accordance with previous description [13]. The New Zealand White was used (Egle Kergiene, Lundsbrunn, Sweden). Briefly, the experiments were performed on a total of 155 rabbit pups from 43 litters (92 females and 63 males) delivered via c.s. after the does were anesthetized with intravenous Propofol-Lipuro (20 mg/ml intravenous (i.v.), B. Braun Melsungen AG, Melsungen, Germany) on day 29 (term 31–32 days). After delivery, the pups were handled and nursed by the animal laboratory staff. The pups were dried and placed in an infant incubator set to 30 °C and 60% humidity. At approximately. 1–2 h of age, the pups were weighed, marked, and hand-fed with bovine colostrum (100 ml/kg/day, Biodane Pharma, Gesten, Denmark) using a 4 French feeding tube (Vygon, Ecouven, France). At 12 h of age, the pups received a mixture (1:1, v/v) of bovine colostrum and Fox Valley 30/50 (Melk voor Dieren, Rotterdam, the Netherlands). From 24 h and onwards, pups received only Fox Valley 30/50. The administered amount of Fox Valley 30/50 was increased every 24 h by 10 ml/kg/day. Feeding at 12, 36 and 60 h of age were reduced to half of the previous corresponding dose, i.e., 50 ml/kg at 12 h of age (corresponding to half of 100 ml/kg given at birth) etc. Pups were gently cleaned once or twice a day, as required to maintain hygiene.

### Experimental setup

**Fig. 1:**
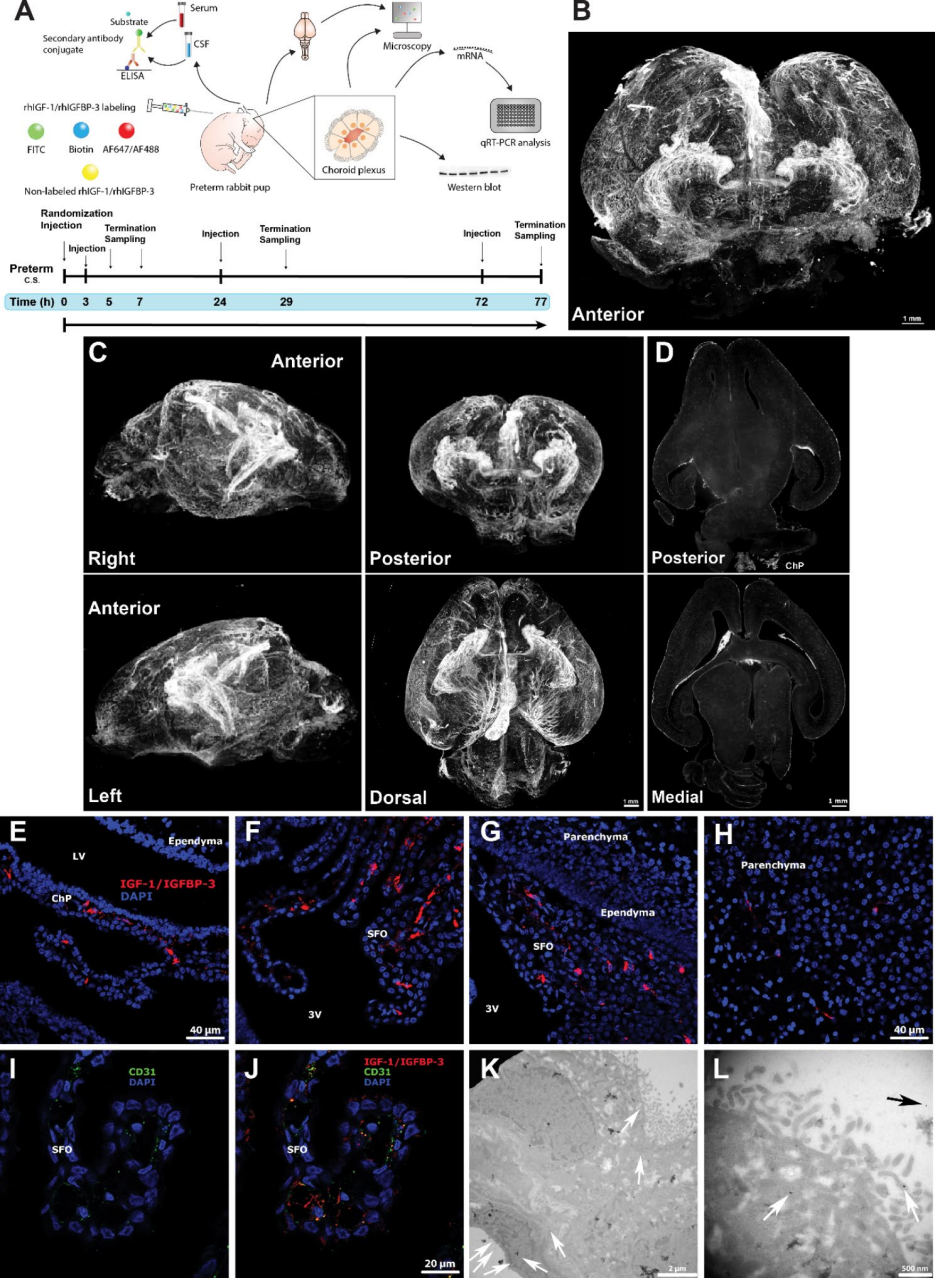
Distribution of rhIGF-1/rhIGFBP-3 in the immature brain of the preterm rabbit pup. **A.** Outline of experimental in vivo setup. **B** and **C.** Representative three-dimensional light-sheet microscopy images illustrating the presence of AF647-labeled rhIGF-1/rhIGFBP-3 (in white) in the ChP, perivascular and subarachnoid space. **D.** Representative two-dimensional light-sheet microscopy images illustrating distinct presence of rhIGF-1/rhIGFBP-3 in the ChP of the lateral and third ventricle (white, **lower**), and to a lesser degree in the fourth ventricle (white, **upper**). Light-sheet images were collected from 4 independent experiments. **E-J.** Representative confocal microscopy images illustrating biotin-labeled rhIGF-1/rhIGFBP-3 (subsequently visualized with streptavidin-coupled AF647) in ChP of the lateral ventricle (E, red), the SFO in the third ventricle (F-G, red), and negligible presence in the parenchyma (G-H, red). **I-J.** Visualization of CD31 at the endothelial lining of the SFO (I, green) and co-existence with rhIGF-1/rhIGFBP-3 (J, CD31 green, rhIGF-1/rhIGFBP-3 red). Cell nuclei were counterstained with DAPI (E-J, blue). Confocal images were collected from 3 independent experiments. **K-L.** Representative TEM images illustrating rhIGF-1/rhIGFBP-3 labeled with FITC (subsequently visualized with anti-FITC gold conjugate) at the basal (**K**) and the apical (**L**) border of the ChP in the lateral ventricles. White and black arrows display gold-labeled rhIGF-1/rhIGFBP-3. TEM images were collected from 2 independent experiments. C.S., cesarean section; GA, gestational age; E, embryonic; P, postnatal; LV, lateral ventricle; SFO, subfornical organ; 3V, third ventricle. For additional experimental details, see Methods and Results section

A schematic illustration of the experimental setup, including techniques used, is outlined in Fig. 1A.

#### Characterization of uptake of s.c. administrated labeled rhIGF-1/rhIGFBP-3 in the immature brain of preterm rabbit pups

At 1–2 h of age, pups were randomized, based on equal mean of weights and litter etc. into respective groups. At approximately 24 h of age, pups received either a s.c. injection of rhIGF1/rhIGFBP-3 labeled with either biotin (n = 9), FITC (n = 2) or Alexa Flour (AF) 647 (n = 4) (8 mg/kg total dose administered, composed of 0.116 mg biotin-, FITC- or AF647-labeled rhIGF-1/rhIGF-BP-3 and 0.1–0.2 mg non-labeled rhIGF-1/rhIGFBP-3, see details in section “Labeling of rhIGF-1/rhIGFBP-3”) or vehicle. Animals were terminated, as described in section “Tissue collection and processing”, 5 h post-injection and the brains were perfused, with paraformaldehyde (PFA), dissected out and subsequently analyzed using transmission electron (TEM), confocal- and light-sheet microscopy (see details in sections “Tissue collection and processing”, “Immunofluorescence microscopy and immunohistochemistry”, “Light-sheet microscopy” and “Electron microscopy”).

#### Characterization of IGF-1 immunoreactivity and IGF-1 concentration in CSF and serum following s.c. administration of unlabeled rhIGF-1/rhIGFBP-3

Immediately after birth, pups were randomized into four groups denoted 0, 3, 24 and 72 h (groups 0, 24 and 72 h, n = 22–24 per group; group 3 h, n = 4), and at respective corresponding time-point, received a s.c. injection of 8 mg/kg of unlabeled rhIGF-1/rhIGFBP-3 (supplied and prepared in vehicle solution as described by Takeda Pharmaceutical Company Ltd, Boston, MA, USA). Pups were followed for 4 (3 h group) or 5 h (0, 24 and 72 h groups), and subsequently terminated and brains were collected for analysis of IGF-1 immunoreactivity (se details in section “Tissue collection and processing”, and “Immunofluorescence microscopy and immunohistochemistry”), ChP tissue was extracted for determining IGF-1R activation and gene expression (see details in section “Tissue collection and processing”, “Protein extraction from rabbit ChP tissue”, “Western blot analysis and RNA isolation and qRT-PCR/gene array analysis”), and CSF and blood was collected to determine IGF-1 concentration in both fluids (see details in section “Tissue collection and processing”, and “IGF-1 concentrations”).

The animals in the 3 h injection group, terminated at 7 h, were obtained from an initial pilot study. Subsequently, a minor adjustment in the study setup was implemented due to experimental conditions. This was not observed to have any significant impact on the data.

In addition to the above described experimental setups, additional pups were used for establishment of experimental procedures, incl. IGF-1R activation, immunohistochemistry, immunofluorescence and immunogold labeling. These pups are not included in the experimental groups, but are included in the total number of animals reported above.

Sex of all pups reported in this work was determined as described in section “Sex determination” using an ears biopsy. The sex determination was performed retrospectively and was not part of the assignment of animals to different experimental groups. Sex distribution was observed to be similar in both total rabbit pups and experimental groups, see further details in Additional File 1: Table S1.

### Labeling of rhIGF-1/rhIGFBP-3

AF647-, biotin- and FITC conjugation of rhIGF-1/rhIGFBP-3 (supplied and prepared in vehicle solution as described by Takeda Pharmaceutical Company Ltd, Boston, MA, USA) was performed using Alexa Fluor® 647 Conjugation Kit (Fast, Lightning-Link®, ab269823, Abcam, Cambridge, UK), Biotin Conjugation Kit (Fast, Type A, Lightning-Link, ab201795, Abcam) and FITC Conjugation Kit (Fast, Lightning-Link, ab188285, Abcam) according to the manufacturer’s instructions. For all labeling protocols, 25% of the recommended protein concentration of rhIGF-1/rhIGFBP-3 was used (according to recommendation from the manufacturer).

### Tissue collection and processing

For Western blot analysis, pups were terminated by decapitation, and blood (for IGF-1 analysis) and ChP was immediately collected. ChP were snap frozen in liquid nitrogen and subsequently transferred to dry ice. For quantitative real-time PCR (qRT-PCR), pups were anaesthetized with an intramuscular (i.m.) injection of Ketaminol vet. (50 mg/ml, Intervet AB, Stockholm, Sweden) and Rompun vet. (20 mg/ml, Bayer Animal Health, Leverkusen, Germany) in combination with isoflurane inhalation (Attane vet, 1000 mg/g, VM Pharma AB, Stockholm, Sweden). Following sedation, CSF (approximately 20–70 µl, for IGF-1 analysis) was sampled from the neck region, fourth ventricle basal cistern, using a 30-gauge syringe, transferred to a 1.5 ml centrifugation tube, visually inspected for stick bleedings (to exclude blood contamination from the insertion of the syringe needle), and centrifuged 1000 xg for 10 min. The supernatant was collected and transferred to a fresh 1.5 ml centrifugation tube, snap frozen, and stored at -80 °C until further analysis as described below. A blood sample (for IGF-1 analysis) was collected through heart puncture, and was centrifuged at 1000 xg for 10 min, for serum and plasma preparation, snap frozen on dry ice and stored at -80 °C until further analysis as described below. An ear biopsy (for sex determination) was collected and snap frozen on dry ice and stored at -80 °C until further analysis as described below. Pups were subsequently transcardially perfused with freshly prepared phosphate buffer (PBS, 0.1 M phosphate buffer, pH 7.4, containing heparin 1000 IU/ml), and the brains were extracted from the skull. The area of interest, the ChP, was collected, snap frozen on dry ice and stored at -80 °C until further analysis as described below (see section “RNA isolation and qRT-PCR/gene array analysis”). For immunohistochemistry (IHC), immunofluorescence (IF), TEM and light-sheet microscopy, rabbit pups were anaesthetized as described above, and subsequently transcardially perfused with freshly prepared PBS (containing heparin 1000 IU/ml), followed by perfusion with freshly prepared 4% PFA (VWR Chemicals, Leuven, Belgium, buffered with PBS, pH 7.4). After fixation, the brains were extracted from the skull and post-fixed by immersion in 4% PFA. A change to fresh PFA was performed after 6–8 h and brains were then immersed in PFA for a total of 24 h at 4 °C, and transferred to PBS for further processing as described in respective section below.

### Immunofluorescence microscopy and immunohistochemistry

PFA-fixated brains were dehydrated in a graded ethanol series (70-99.99%) ended with xylene (100%), and subsequently immersed in paraffin and embedded in paraffin blocks. Coronal and sagittal paraffin sections (4 or 6 μm) were prepared on a rotating microtome (Microm HM 360, Microm International GmbH, Walldorf, Germany). For freeze embedding, PFA-fixated brains were rinsed in PBS (pH 7.4), and subsequently immersed in anti-freeze solution (sucrose 10 and 20%) before frozen in molds with OCT (Histolab, Gothenburg, Sweden). Cryosections (10 μm) were prepared on a cryotome (Leica CM3050 S, Wetzlar, Germany). All sections were collected on microscope slides (SuperFrost Plus, Thermo Scientific/Gerhard Menzel B.V. & Co., Braunschweig, Germany).

For IF double labeling of IGF-1R and IGF-1, de-paraffinized and rehydrated sections were rinsed in PBS containing 0.05% Triton X-100 (TX), followed by blocking in PBS-TX, and 1% bovine serum albumin (BSA, PBS-TX-BSA) for 30 min at room temperature. Sections were then incubated with a cocktail of primary antibodies against IGF-1R (goat polyclonal IgG, 2 µg/ml, diluted in PBS-TX-BSA, AF-305-NA, R&D System, McKinley Place, MN, USA) and IGF-1 (mouse monoclonal IgG, 2 µg/ml, diluted in PBS-TX-BSA, AM33345PU-S, Origene, Herford, Germany) for 16 h at 4 °C. Following rinses in PBS-TX, sections were incubated for 45 min at room temperature with a mixture of AF488 conjugated secondary antibodies against goat IgG (IGF-1R) and RhodamineRX-conjugated antibodies against mouse IgG (IGF-1, both from Jackson ImmunoResearch, West Grove, PA, USA, diluted 1:200 in PBS-TX). Sections were then rinsed in PBS-TX and incubated in 4′,6-diamidino-2-phenylindole (DAPI) for 15 min at room temperature, followed by a rinse in PBS and mounting in anti-fade solution (Abcam). Control sections were used to set the background threshold level of fluorescence detection levels for the confocal laser scanning analysis and imaging (Zeiss LSM 800, Zeiss, Dresden, Germany) using x20 dry or x40 oil immersion lenses. Sequential scanning was performed, with optimized pinhole size for each channel. For detection of in vivo administrated biotin- and FITC-conjugated rhIGF-1/rhIGF1BP-3, cryosections from injected and non-injected animals were incubated with Streptavidin AF647 (JIR 15,095, Jackson ImmunoResearch, 2 µg/ml, diluted in PBS-TX) for 45 min at room temperature, or with anti-FITC (mouse monoclonal IgG, ab10257, Abcam, 5 µg/ml, diluted in PBS-TX) for 60 min at room temperature. For visualization of FITC/anti-FITC, sections were incubated with anti-mouse IgG conjugated with AF488 (Jackson ImmunoResearch, diluted 1:200 in PBS-TX) for 30 min at room temperature. For double labeling of IGF-1 or cluster of differentiation 31 (CD31) together with biotin- and FITC-conjugated rhIGF-1/rhIGF1BP-3, cryosections were incubated with primary antibodies against IGF-1 (as described above, 4 µg/ml, diluted in PBS-TX) or CD31 (AF3628, R&D, 7 µg/ml, diluted in PBS-TX), for 60 min at room temperature. IGF-1 was visualized as above, and CD31 was visualized by incubation with AF488-conjugated secondary antibodies (donkey anti-goat, Jackson ImmunoResearch 705-546-147, diluted 1:200 in PBS-TX). For simultaneous visualization of biotin-conjugated rhIGF-1/rhIGF1BP-3, and the immunolabeling of IGF-1 or CD31, labelled sections were incubated with Streptavidin AF647. Confocal laser scanning microscopy was performed as above, using threshold settings for detection levels obtained in sections from non-injected animals and in negative control sections (excluded primary antibody incubation of parallel sections).

For chromogen visualization of IGF-1 immunoreactive sites, nickel ion intensification of the immunoreaction was used. Sections were de-paraffinized and rehydrated, starting in xylene followed by a graded ethanol series ending in PBS (pH 7.4). Sections (4–6 μm) were then quenched for endogenous peroxidase activity, in hydrogen peroxidase (0.1%) for 10 min, followed by incubation in PBS-TX-BSA for 30 min, both at room temperature. Sections were then incubated with IGF-1 primary antibody (as described above, 2 µg/ml, diluted in PBS-TX-BSA) for 16 h at 4 °C. Following rinses in PBS-TX, sections were incubated with horseradish peroxidase (HRP)-conjugated secondary antibodies (goat anti-mouse IgG, K4001, DAKO Envision, Agilent, Santa Clara, CA, USA, diluted 1:1 in PBS-TX-BSA), for 30 min at room temperature. The HRP conjugation was reacted in a PBS solution containing diaminobenzidine (0.5 mg/ml, Sigma D5637, Merck, Solna, Sweden), hydrogen peroxidase (0.1%, Millipore 107,298, Merck, Solna, Sweden) and nickel ions (0.125% ammonium nickel(II) sulphate hexahydrate, A1827, Merck, Solna, Sweden), for 10 min at room temperature. Sections were then rinsed in PBS, dehydrated (graded ethanol ending with 100% xylene), mounted (Pertex, Histolab, Gothenburg, Sweden), and cover slipped. Chromogen labeled coronal and sagittal sections were analyzed and imaged in a bright-field microscope (Olympus IX73, Shinjuku, Tokyo, Japan).

All antibody incubations were performed in a moisture chamber. The specific of the labeling against IGF-1R and IGF-1 was confirmed by the distribution of labeling in rabbit and human placenta tissue (i.e. positive control tissue), including omitting (in all runs) the primary antibody incubation (negative control), and by a corresponding labeling pattern in the rabbit brain to previous reports [3]. CD31 antibody specificity was confirmed when omitting the antibody incubation, and the labeling of capillary endothelial cells. Specific detection of administered biotin- and FITC-conjugated rhIGF-1/rhIGF1BP-3 was established by using confocal microscopy threshold settings that only visualized strong signals (above background levels), obtained from non-biotin and non-FITC conjugated rhIGF-1/rhIGF1BP-3 treated animals and negative control sections of non-treated and treated animals.

### Light-sheet microscopy

Extracted PFA-fixated brains were transferred to graded methanol series ending in PBS (pH 7.4, 1 h per step, followed by 100% methanol overnight). Brains were thereafter incubated in 67% dichloromethane (DCM, Merck, Solna, Sweden) and 33% methanol for 24 h (with shaking), followed by 2 × 15 min wash in 100% DCM (with shaking). Subsequently, the brains were incubated in ethyl cinnamate (EtCinn, Merck, Solna, Sweden) for 24 h. Lastly, the brains were stored in fresh EtCinn. Light-sheet microscopy was conducted using an ultramicroscope blaze light-sheet microscope (Miltenyi Biotec, Bergisch Gladbach, Germany).

### Electron microscopy

Extracted rabbit ChP were fixed in 2.5% glutaraldehyde (Merck) in 0.15 M sodium cacodylate (pH 7.4, Sigma-Aldrich, Steinheim, Germany), embedded in Epon (Agar Scientific Ltd, Stansted, Essex England) and ultrasectioned. Samples were subjected to antigen retrieval with sodium metaperiodate and subsequently incubated with primary anti-FITC antibodies (mouse monoclonal IgG, Abcam, ab10257, 1 ug/ml, diluted in 0.2% Aurion BSA (AURION, Wageningen, the Netherlands) in PBS pH 7.6.) followed by detection with species-specific secondary antibody-gold conjugates (EM goat anti-mouse IgG 15 nm Gold, BBInternational, Cardiff, UK). The sections were stained with uranyl acetate (0.5%, Laurylab, Saint Fons, France) and lead citrate (3%, Laurylab). Rabbit ChP were examined in a FEI Technai Biotwin 120kv TEM operated at 100 kV accelerating voltage. Images were recorded with side-mounted Olympus Veleta camera with a resolution of 2048 × 2048 pixels (FEI, Hillsboro, OR, USA).

### Establishment of murine primary ChP epithelial cell culture

The animal protocols were approved by the Swedish Animal Ethics Committee in Lund, Sweden (Dnr. 5.8.18–12,930/2019). Primary murine ChP epithelial (ChPE) cell culture studies were performed as previously described [14]. Briefly, ChP from the lateral and fourth ventricle was isolated from postnatal day 3–6 murine pups (C57Bl/6Ncrl, Scanbur, Karlslunde, Denmark) under a dissection microscope (Nikon SMZ800N Stereomicroscope, Tokyo, Japan). Collected ChPE cells were digested using the enzymatic reaction of 2 mg/ml pronase (isolated from Streptomyces griseus, Merck, Burlington, MA, USA). The digestion was terminated with the addition of excess complete DMEM-F12 cell culture medium (Gibco, Waltham, MA, USA) containing 10% fetal bovine serum (FBS, Gibco) and 1% Antibiotic-Antimycotic (Gibco). The cells were thereafter centrifuged 1000 xg for 2 min, resuspended in completed DMEM-F12 culture medium and plated (2*10^5^ cells/well) on a 24-well plate (Fisher Scientific, Gothenburg, Sweden). To ensure minimal fibroblast contamination, cell culture media was changed after 48 h to complete DMEM-F12 culture media containing cytosine arabinoside (Ara-C, Merck). The complete DMEM-F12 culture medium was thereafter changed every 48 h until ChPE cells reached approx. 90% confluence, taking 8–10 days. Cells were cultured at 37 °C in 5% CO_2_.

### Treatment and lysis of ChPE cell cultures

When ChPE cell cultures reached approximately 90% confluence, the cells were serum starved for 1–2 h prior to exposure with rhIGF-1/rhIGFBP3, rhIGF-1 or control, by changing the cell culture media to DMEM-F12 culture media with 0% FBS. Subsequently, the cells were washed with serum free cell culture media, and then incubated for 15 min, 2 and 3.5 h at 37 °C in 5% CO_2_, with rhIGF-1/rhIGFBP3 (300 ng/ml, corresponding to 60 ng/ml IGF-1, prepared in DMEM-F12 cell culture media, supplied by Takeda Pharmaceutical Company Ltd, Boston, MA, USA), rhIGF-1 (60 ng/ml, prepared in DMEM-F12 cell culture media, supplied by Takeda Pharmaceutical Company Ltd, Boston, MA, USA) or FMEM-F12 cell culture media only (control). For protein analysis, the cells were washed with ice cold PBS (pH 7.4), incubated in 60 µl lysis buffer (1 mM EDTA, 1 mM EGTA, 5 mM Na-pyro-phosphate, 0.27 M sucrose, 50 mM NaF, 50 mM Tris-Base, 1 mM Na-ortho-vanadate and 1% w/v NP40) containing freshly added protease inhibitor cocktail and 1 mM dithiothreitol (DTT), scraped, transferred to a centrifugation tube and centrifuged at 15,000 xg for 15 min at 4 °C. Subsequently, the supernatant was transferred to a new tube and stored at -80 °C until further analysis as described below. For total RNA extraction, cells were collected by incubation in 300 µl QIAzol (Qiagen, Alden, Germany), and further processed according to manufacturer’s instructions.

### RNA isolation and qRT-PCR/gene array analysis

RNA was isolated from rabbit ChP tissue utilizing the RNeasy Midi Kit (Qiagen). RNA purity and concentration was determined using Qubit Fluorometric Quantification (Thermo Fisher Scientific, Waltham, MA, USA). 500 ng of RNA was used as a template for reverse transcription reactions, performed according to the instructions from the manufacturer using the iScript RT kit (Bio-Rad, Hercules, CA, USA). Gene expression analysis, using Primer PCR assay from Bio-Rad, of transthyretin (TTR, qOcuCID0013554), orthodenticle homeobox 2 (OTX2, qOcuCED0010795), bone morphogenetic protein 4 (BMP4, qOcuCED0010761), folate receptor 1 (FOLR1, qOcuCID0014000), albumin (ALB, qOcuCID0005559), bone morphogenetic protein 7 (BMP7, qOcuCED0016395), aquaporin-1 (AQP1, 330,001), aquaporin-5 (AQP5, qOcuCED0015146), and transforming growth factor beta (TGF- β, qOcuCID0002132) was performed by mixing cDNA with iTaq Universal SYBR Green Supermix (Bio-Rad). Amplification was performed as described by the manufacturer (Bio-Rad) for 40 cycles in a CFX Connect thermal cycler (Bio-Rad), and data were analyzed using CFX Maestro Software (Bio-Rad). Data were normalized against hypoxanthine phosphoribosyltransferase 1 (HPRT, qOcuCID0004422) and the 2 − ΔΔCT method was used to determine fold change expression, by normalizing against ChP from 0 h aged untreated pups.

RNA was isolated from primary murine ChPE cells utilizing the RNeasy Mini Kit (Qiagen), RNA purity and concentration was determined using Qubit Fluorometric Quantification (Thermo Fisher Scientific).

The expression of 84 genes related to angiogenesis (RT2 Profiler PCR Array, Mouse Angiogenesis, PAMM-024Z, Qiagen) was evaluated in all experimental conditions by using 1 µg of RNA as template for the reverse transcription reactions (RT2 First Strand Kit. Qiagen). RT2 SYBR Green qPCR Master mix (Qiagen) was used to quantify the mRNA expression. Amplification was performed as described by the manufacturer (Bio-Rad) for 40 cycles in a CFX Connect thermal cycler (Bio-Rad), and data were analyzed using CFX Maestro Software (Bio-Rad). Data were normalized to β-actin (ActB) and glyceraldehyde 3-phosphate dehydrogenase (GAPDH), both included in the RT2 Profiler Array, and the 2 − ΔΔCT method was used to determine fold change expression by normalizing against ChPE cells from untreated cells.

### IGF-1 concentrations

Serum and CSF concentration of IGF-1 was determined using the Human IGF-1 ELISA (Mediagnost, Reutlingen, Germany) according to the manufacturer’s instructions.

### Protein extraction from rabbit ChP tissue

An ice cold steel bead (Qiagen) was placed in a sample tube with ChP tissue on dry ice. 180 µl of cold complete lysis buffer (described above in section “Exposure of ChPE cell cultures”) containing a protease inhibitor cocktail and 1 mM DTT. Tissue was disrupted using a TissueLyser LT 2 (Qiagen) for 2 min at 50 Hz, and thereafter incubated on ice for 5 min. Subsequently, samples were centrifuged 15 000 xg for 15 min at 4 °C. The supernatant was transferred to a new tube and stored at -80 °C until further analysis as described below.

### Western blot analysis

Sodium dodecyl-sulfate polyacrylamide gel electrophoresis (SDS-PAGE) was conducted as previously described [15] using pre-cast stain-free 4–20% gels (Mini-Protean TGX, Bio-Rad) on samples mixed with sample buffer (Bio-Rad) and heat denatured. Protein size was determined with Precision Plus Protein All Blue Prestained Protein Standards (Bio-Rad). Gels were subjected to electroblotting (Transblot® Turbo, Bio-Rad), and after transfer to polyvinylidene difluoride (PVDF) membranes, membranes were incubated in blocking solution (5% non-fat dry milk, Bio-Rad, in PBS containing 0.05% Tween, PBS-T), followed by a primary mouse phospho-AKT/PKB antibody (Proteintech, 66444-1-Ig, Rosemont, IL, USA, diluted 1:2000 in 5% non-fat dry milk in PBS-T), or phospho-ERK1/2 (Invitrogen, 14-9109-82, Waltham, MA, USA, diluted 1:500 in 5% non-fat dry milk in PBS-T), or a primary rabbit phospho-AKT/PKB (Thermo Fisher, 44-621G), phospho-ERK1/2 (Cell Signaling Technology, 9102, Danvers, USA). All rabbit primary antibodies were diluted 1:1000 in 5% non-fat dry milk in PBS-T. Goat anti-mouse IgG HRP (Dako, Glostrup, Denmark; for mouse primary antibodies), or swine anti-rabbit IgG HRP (Dako; for rabbit primary antibodies), both diluted 1:1700 in 1% non-fat dry milk in PBS-T, was used as secondary antibodies. Clarity Western ECL Substrate (Bio-Rad) was used to detect signals from HRP-conjugates. Re-blotting against β-actin was performed by using a primary mouse anti-actin antibody (Abcam, ab6276) or primary rabbit anti-actin antibody (Abcam, ab213262), both diluted 1:5000 in 1% non-fat dry milk in PBS-T. Goat anti-mouse or swine anti-rabbit IgG-HRP (Dako, diluted 1:1700 in 1% non-fat dry milk in PBS-T) was used as secondary antibodies. Signals from HRP-conjugates were detected using Clarity Western ECL Substrate (Bio-Rad). Membranes and gels were imaged and analyzed using the ChemiDoc™ MP System (Bio-Rad).

### Liquid chromatography–mass spectrometry

Samples, 60 µl, prepared as described above “Treatment and lysis of ChPE cell cultures”, were reduced with dithiothreitol to a final concentration of 10 mM and heated at 56 °C for 30 min followed by alkylation with iodoacetamide to a final of 20 mM for 30 min at room temperature in dark. Samples were precipitated with ice cold ethanol (final concentration of ethanol 90%) overnight at -20 C followed by centrifugation at 14 000 x g for 10 min. The pellets were air dried and resuspended with 100 µl of 100 mM ammonium bicarbonate and sonicated using a Bioruptor (Diagenode), 30 cycles (20 s on, 20 s off). Protein concentration was measured at 280 nm using a NanoDrop (DeNovix DS-11, DeNovix Inc; Wilmington, DE, USA). Digestion was performed by adding trypsin in a ratio of 1:50 (Sequencing Grade Modified Trypsin, Part No. V511A, Promega) to the samples and incubation overnight was performed at 37 °C. The digestion was stopped by 5 µl 10% trifluoroacetic acid. The samples were Speed Vac to dryness and resolved in 2% ACN, 0.1%. Peptides extracted were analyzed on an Exploris 480 mass spectrometer (Thermo Fischer Scientific) coupled with a Vanquish Neo UHPLC system (Thermo Fischer Scientific). A Two-column setup was used on the HPLC system, and peptides were loaded into an Acclaim PepMap 100 C18 pre-column (75 μm x 2 cm, Thermo Fisher Scientific) and then separated on an EASY spray column (75 μm x 25 cm, C18, 2 μm, 100 Å, ES902) with the flow rate of 300 nl/minute. The column temperature was set to 45 °C. Solvent A (0.1% FA in water) and solvent B (0.1% FA in 80% acetonitrile) were used to create a 120 min non-linear gradient from 5 to 25% of solvent B for 100 min, increased to 32% for 12 min, and then increased to 45% for 8 min to elute the peptides. The samples were analyzed with a data-dependent acquisition (DDA) in positive mode. The full mass spectrometry spectra 1 (MS1) resolution was set to 120 000 at m/z 200 and the normalized AGC target was set to 300% with the maximum injection time of 45 mseconds. The full mass range was set to 350–1400 m/z. Precursors were isolated with the isolation window of 1.3 m/z, and fragmented by HCD with the normalized collision energy of 30. Mass spectrometry spectra 2 (MS2) was detected in the Orbitrap with the resolution of 15 000. The normalized AGC target and the maximum injection time were set to 100% and custom, respectively. The intensity threshold for precursor selection was set to 1e4 and 60 s dynamic exclusion was applied.

### Data analysis of mass spectrometry data

The raw DDA data were analyzed with Proteome Discoverer™ 2.5 Software (Thermo Fisher Scientific), and the peptides were identified using SEQUEST HT against UniProtKB Mouse canonical database (UP000000589) and fasta files for the human IGF-1 (P05019) and IGFBP-3 (P17936). The search was performed with the following parameters applied: carbamidomethylation of cysteine as static modification, N-terminal acetylation, and methionine oxidation as dynamic modification. Precursor tolerance was set to 10 ppm and fragment tolerance was set to 0.02 ppm. Up to 2 missed cleavages were allowed and Percolator was used for peptide validation at a q-value of maximum 0.01. Extracted peptides were used to identify and quantify them by label-free relative quantification. The extracted chromatographic intensities were used to compare peptide abundance across samples.

Normalization of the peptides intensities was conducted with the R-package NormalyzerDE [16]. Median normalized values were used. Missing values were filtered to that each protein had a minimum of four 4 values in each group. Pairwise comparisons between treatments were performed on the summed peptide abundance over all treatment groups. The differential expression of proteins was analyzed using a linear model with the sample group, protein type, and their interaction as independent variables. Contrasts between groups (rhIGF-1/IGFBP-3 and rhIGF-1 vs. control) were specified using the emmeans package [17], then log2-transformed and subsequently reported with 95% confidence intervals. Adjusted p-values were calculated using the Benjamini-Hochberg method with a q-value of 0.05. Additionally, proteins with a 95% confidence interval not spanning zero were considered differentially abundant. Enrichment analysis was performed by searching the Gene Ontology (GO), David and STRING databases for Reactome pathways, and Kyoto Encyclopedia of Genes and Genomes (KEGG) pathways [18–20].

### Sex determination

Determinations of rabbit sex were performed by confirming the presence of the sex-determining region Y gene (gene ID: 100,328,958) in the rabbit genome using PCR and gel electrophoresis visualization as described before [3]. Briefly, DNA was extracted from an ear biopsy using DNeasy Blood and Tissue Kit (Qiagen) according to the manufacturer’s instructions. One µl of DNA (range: 100–200 ng/µl) was used in the PCR reaction (30 cycles at 57 °C) with the following primers: Sense: TGCAATACAGGAGGAACACG, Antisense: AGCAAACTGTCGCTCTTCTG. Presence of a band at approximately 299 bp determined the animal as male, and no corresponding band determined the animal as female.

### Statistics

Statistical significance was calculated using one-way ANOVA with a post hoc Tukey for multiple comparisons of means, or Student’s t-test for pair-wise comparisons. P-values < 0.05 were considered significant. Data is presented as means ± SD (see also respective figure legend for additional details). Statistical analyses were performed using R version 4.0.3.

## Results

### Distribution of rhIGF-1/rhIGFBP-3 in the immature brain upon s.c. administration

We performed biotin-, FITC- and AF647 conjugation of the rhIGF-1/rhIGFBP-3 complex and subjected preterm rabbit pups to s.c. injection of 8 mg/kg of a mixture of conjugated and non-labeled rhIGF-1/rhIGFBP-3 (0.116 mg biotin-, FITC- or AF647-labeled and 0.1–0.2 mg non-labeled adjusted based on weight). RhIGF-1/rhIGFBP-3 was administered at a postnatal age of 0, 3, 24 and 72 h, and terminated 4–5 h post-administration, i.e. corresponding to 5, 7, 29 and 77 h of postnatal age. A schematic illustration of the experimental setup, including techniques used, is outlined in Fig. 1A.

Following termination, the rabbit pup brains were extracted, and prepared for visualization of IGF-1 immunoreactivity. We found increased IGF-1 immunoreactivity in and adjacent structures to the ChP, including surrounding parenchyma, hippocampus and cerebellum in 7 h old preterm rabbits upon administration of unlabeled rhIGF-1/rhIGFBP3, compared to vehicle (Additional file 2, Fig. S1 1 A-D). To determine if the increase in IGF-1 immunoreactivity was caused by endogenous production, or an uptake of the administered rhIGF-1/rhIGFBP-3, we investigated the distribution of IGF-1 following s.c. administration of AF647-labeled rhIGF-1/rhIGFBP-3 in the brains of 24 h aged pups, using light-sheet microscopy. RhIGF-1/rhIGFBP-3 was primarily present in ChP of the lateral and third ventricles, the perivascular space and in the subarachnoid space 5 h post s.c. administration (Fig. 1B-D, white). Of note, rhIGF-1/rhIGFBP-3 was observed to a lesser extent in the fourth ventricle (Fig. 1D). A video rotating horizontally can be seen in Additional file 3, Movie. S1. The presence of rhIGF-1/rhIGFBP-3 in the lateral and third ventricles was further confirmed by confocal microscopy (Fig. 1E-F, red). Moreover, rhIGF-1/rhIGFBP3 -1 was observed to be present at the endothelial lining of the ChP, both in the lateral ventricle and in the subfornical organ (SFO), with negligible presence in the parenchyma (Fig. 1G-H, red). In addition, the endothelial marker CD31 was shown to co-exist with rhIGF-1/rhIGFBP3 at the endothelium of the SFO (Fig. 1I-J). To be able to visualize a putative translocation of rhIGF-1/rhIGFBP3 through the ChP, TEM analysis was conducted. Using immunogold labeled antibodies towards FITC-conjugated rhIGF-1/rhIGFBP-3, signal was primarily observed at the endothelial barrier of ChP (Fig. 1K, white arrows), however, with higher magnification, immunolabeling was also observed within epithelial cell villi suggesting translocation of rhIGF-1/rhIGFBP-3 through the ChP (Fig. 1L, white and black arrows).

### Systemic and CSF uptake of IGF-1 in preterm rabbit pups following s.c. administration of rhIGF-1/rhIGFBP-3

**Fig. 2:**
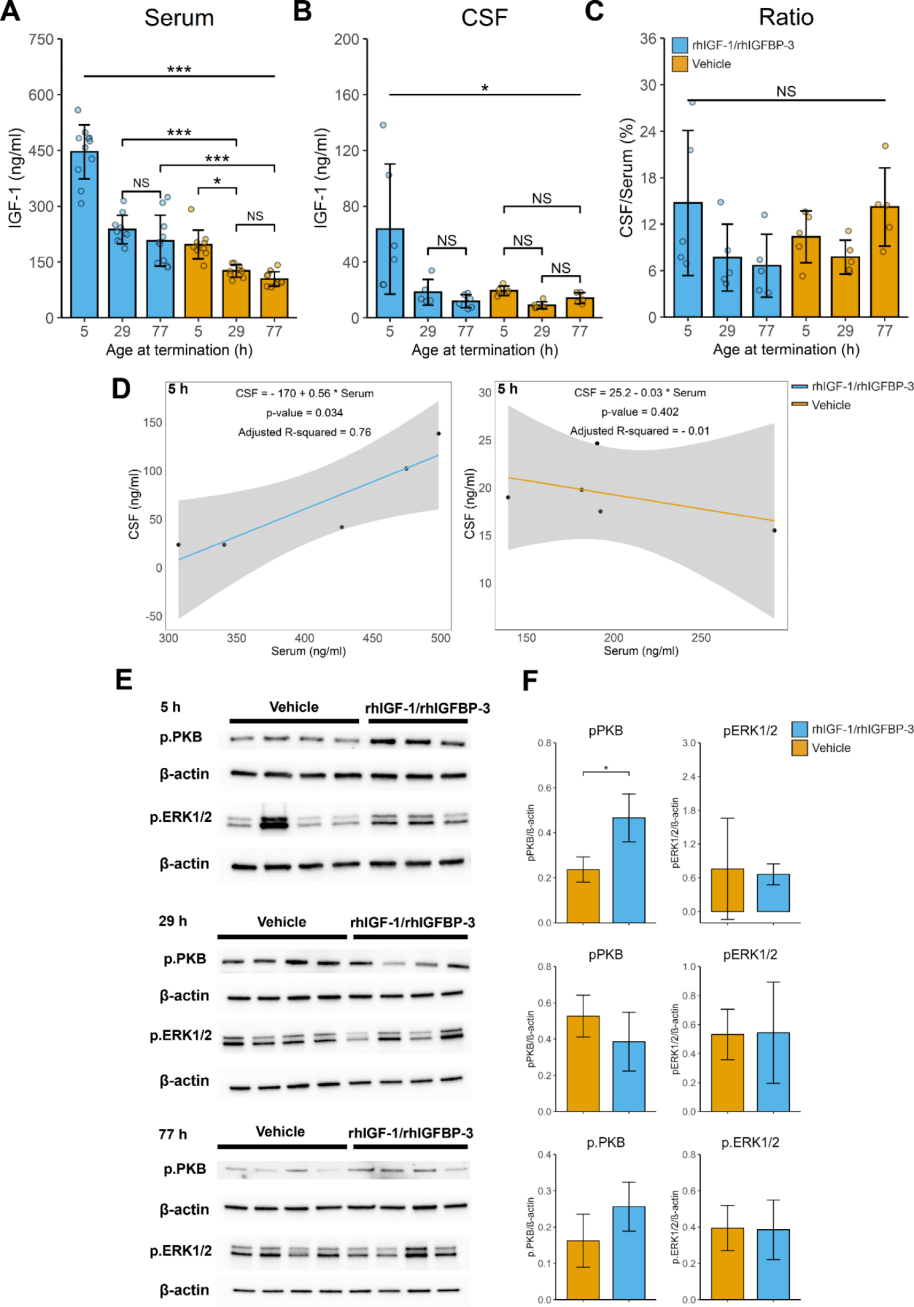
Impact of postnatal age on serum and CSF levels of IGF-1, and activation of IGF-1R in the ChP of the immature brain. **A-D**. Levels of IGF-1 in serum (**A**) and CSF (**B**) in preterm rabbit pups, evaluated 5 h after s.c. injections of rhIGF-1/rhIGFBP-3 (8 mg/kg) or the corresponding vehicle solution at 0 (5), 24 (29) or 72 (77) hours postnatal age (corresponding time-point for termination within parentheses). The ratio of CSF IGF-1/serum IGF-1 was calculated (**C**). Linear model displaying the relation between the IGF-1 levels in serum and CSF in 0 (5) hours postnatal age pups (corresponding time-point for termination within parentheses) (**D**). Dark grey area represents the 95% confidence level. Data are presented as means ± SD (N = 6–9). Differences in IGF-1 concentrations were analyzed using one-way ANOVA with post hoc Tukey test for multiple comparisons of means. *P ≤ 0.05, **P ≤ 0.01, ***P ≤ 0.001. **E**-**F.** Western blot analysis of pERK1/2 and pPKB in ChP from preterm rabbit pups 5 h after s.c. administration of rhIGF-1/rhIGFBP-3 (8 mg/kg) at 0 (5, **upper**), 24 (29, **middle**) or 72 h (77, **lower**) postnatal age (corresponding time-point for termination within parentheses). Data from 3 different experiments are presented as means ± SD (N = 3–4). A representative blot is shown. Differences between groups were analyzed using Student’s t-test. *P ≤ 0.05; NS, not significant

Next, we evaluated the IGF-1 concentration in serum and CSF following s.c. administration of rhIGF-1/rhIGFBP-3 or vehicle. The levels of IGF-1 obtained in serum and CSF were found to vary depending on the postnatal age of the preterm rabbit pup at the time of rhIGF-1/rhIGFBP-3 administration. Administration at a postnatal age of 0 h, i.e. at embryonic day E29, resulted in a significantly higher serum and CSF IGF-1 level than administration at a postnatal age of 24 and 72 h, i.e. corresponding to E30 and E31 (also corresponding to postnatal day P0) (Fig. 2A and B). In line with previous studies [3], we observed that the endogenous serum IGF-1 levels (Fig. 2A, orange bars) decreased over time, with a significant reduction between 0 (termination at 5 h) and 24 h (termination at 29 h) postnatal age. However, we did not observe any significant change in endogenous IGF-1 concentration in CSF during the corresponding period (Fig. 2B, orange bars). The ratio of IGF-1 concentration between CSF and serum displayed no difference in relation to postnatal age, neither in pups receiving exogenous rhIGF-1/rhIGFBP-3 nor in pups receiving vehicle. Linear regression analysis investigating the correlation between IGF-1 concentration in serum and CSF showed a positive correlation in pups administered s.c. with rhIGF-1/rhIGFBP-3 at a postnatal age of 0 h (termination at 5 h) (Fig. 2D, left panel). No correlation was observed in the other treatment groups, independent if the pups were treated with exogenous rhIGF-1/rhIGFBP-3 or vehicle (Fig. 2D, right panel, Additional File 2, Fig. S2).

### IGF-1R activation in ChP of preterm rabbit pups following administration of rhIGF-1/rhIGFBP3

We have previously shown that the IGF-1R is abundantly expressed in the ChP, especially on the apical side [3]. This was corroborated here as co-labeling of IGF-1R (red) with the endothelial marker CD31 (green) (Additional File 2: Fig. S3).

Considering that the translocation of IGF-1, from the circulatory compartment to the CSF, appears to be influenced by postnatal age of the preterm rabbit pup, we aimed to investigate if IGF-1R activation followed a similar pattern. Following exposure to either rhIGF-1/rhIGFBP-3 (8 mg/kg) or the corresponding vehicle solution, at 0, 24 or 72 h postnatal age, the preterm rabbit pups were terminated 5 h post-administration, corresponding to 5, 29 and 77 h postnatal age, and ChP tissue was extracted from the lateral ventricles. Subsequently, the extracted ChP tissue was subjected to Western blot analysis of activating phosphorylation of the IGF-1R signaling pathway proteins ERK (pERK1/2), a mitogen-activated protein kinase, and PKB/Akt (pPKB), in the phosphoinositide-3-kinase cascade. Increased band intensity of both pERK1/2 and pPKB was observed at 5 h postnatal age following rhIGF-1/rhIGFBP-3 administration, as compared to vehicle-injected pups (Fig. 2E-F, upper panel). However, no statistically significant difference was recorded for pERK1/2 due to a high signal in one of the vehicle animals. Furthermore, no difference was observed at 29 or 77 h postnatal age, either in pPKB or pERK1/2 (Fig. 2E-F, middle and lower panel). Full Western blot membranes are provided in Additional File 2: Fig. S4.

### Gene expression in the ChP of preterm rabbit pups following administration of rhIGF-1/rhIGFBP-3

**Fig. 3:**
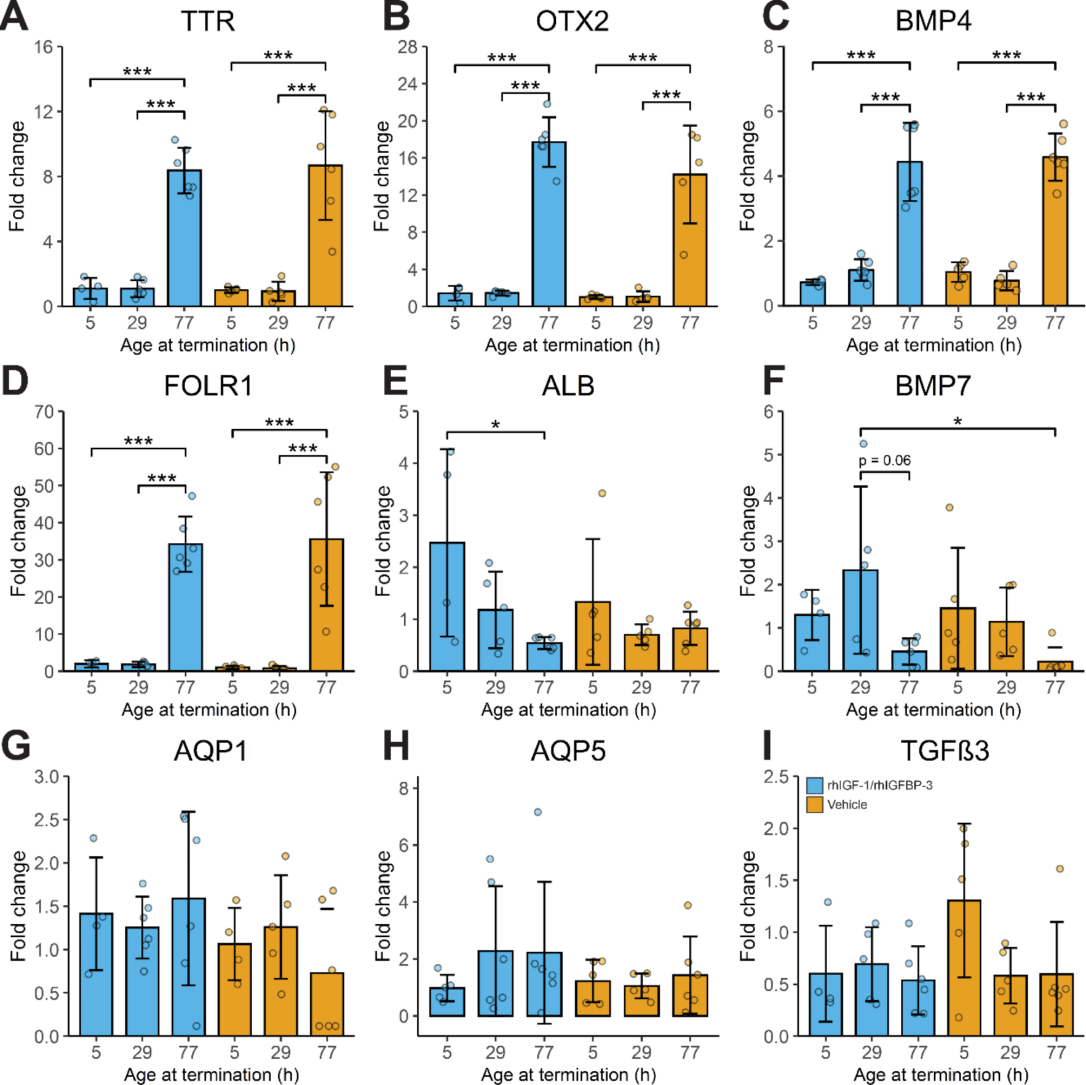
Temporal difference in gene expression in the ChP of preterm rabbit pups. **A-I.** QRT-PCR analysis of TTR (**A**), OTX2 (**B**), BMP4 (**C**), FOLR1 (**D**), ALB (**E**), BMP7 (**F**), AQP1 (**G**), AQP5 (**H**) and TGFβ3 (**I**) in ChP of preterm rabbit pups 5 h after s.c. administration of rhIGF-1/rhIGFBP-3 (8 mg/kg) or the corresponding vehicle solution at 0 (5), 24 (29) or 72 (77) postnatal age (corresponding time-point for termination within parentheses). Data are presented as means ± SD (N = 5–7). Differences in gene expression were analyzed using one-way ANOVA with post hoc Tukey test for multiple comparisons of means, *P ≤ 0.05, ***P ≤ 0.001

The ChP is considered to be under development in rabbit pups born preterm at E29, corresponding to a human gestational age of approx. 24–28 weeks [10, 21]. In the current study, we investigated if the gene expression of maturational relevant targets was altered in ChP following administration of rhIGF-1/rhIGFBP-3 (8 mg/kg). Overall, no major effect was observed following rhIGF-1/rhIGFBP-3 administration as compared to vehicle solution (Fig. 3A-I). However, a minor upregulation, although not significant, of the FOLR1 was observed at 0 and 24 h as compared to vehicle-administered animals at corresponding postnatal ages (Fig. 3D). Notably, genes crucial for the development of the ChP and the brain, including TTR [22], OTX2 [23], BMP4 [24, 25] and FOLR1 were significantly upregulated at 72 h postnatal age, compared to 0 and 24 h postnatal age, independently of rhIGF-1/rhIGFBP-3 administration (Fig. 3A-D). Genes not altered either upon rhIGF-1/rhIGFB-3 administration, or in relation to postnatal age were AQP1, AQP5 and TGFβ3 (Fig. 3G-I).

### Pathway activation, gene expression and protein synthesis in murine ChPE cell cultures following exposure to rhIGF-1/rhIGBP-3 or rhIGF-1

**Fig. 4:**
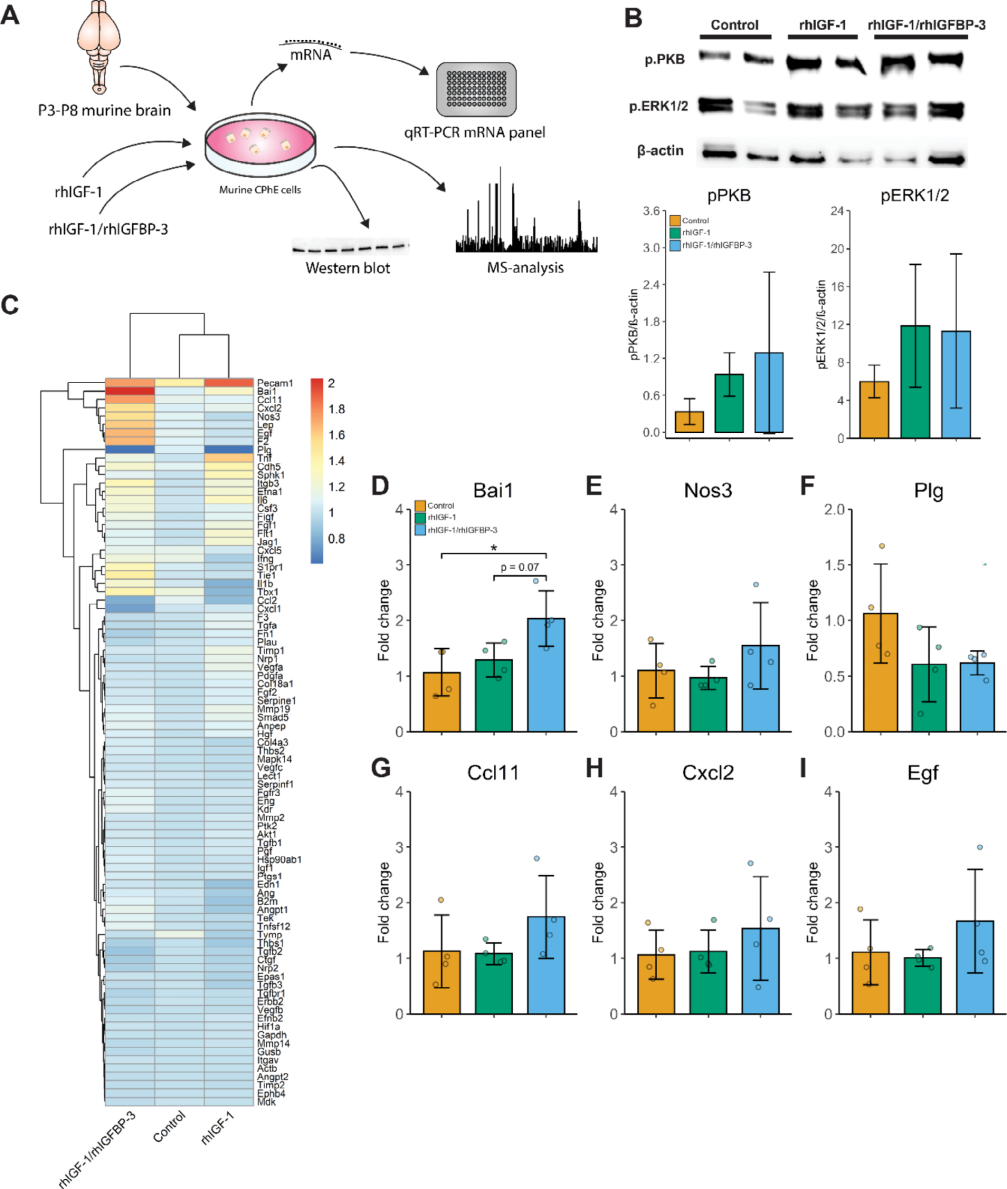
Angiogenesis-related gene expression and IGF-1R activation in ChPE cells upon stimulation with rhIGF-1/rhIGFBP-3 or rhIGF-1. **A.** Outline of experimental in vitro setup. **B.** Western blot analysis of pERK1/2 and pPKB in primary murine ChPE cells following exposure to rhIGF-1/rhIGFBP-3 (300 ng/ml), rhIGF-1 (60 ng/ml) or cell culture media only (Control) for 15 min. Data from are presented as means ± SD (N = 4, pooled samples). **C.** Heat map, showing respective group mean absolute fold change and hierarchical clustering (N = 3–4), comparing gene expression of 84 genes related to angiogenesis using RT2 Profiler PCR Array in primary murine ChPE cells upon stimulation with rhIGF-1/rhIGBP-3 (300 ng/ml), rhIGF-1 (60 ng/ml) or culture medium only (Control) for 2 h. **D - I.** Individual analysis of selected target genes from the RT2 Profiler PCR Array, Bai1 (**D**), nitric oxide synthase 3 (Nos3) (**E**), plasminogen (Plg) (**F**), C-C motif chemokine 11 (Ccl11) (**G**), chemokine (C-X-C motif) ligand 2 (Cxcl2) (**H**) and epidermal growth factor (Egf) (**I**). Data are presented as means ± SD (N = 4). Differences in gene expression were analyzed using one-way ANOVA with post hoc Tukey for multiple comparisons of means, *P ≤ 0.05. P3-P8, postnatal day 3–8

To further characterize the effects of and to confirm the IGF-1R activation by rhIGF-1/rhIGFBP-3, by comparing IGF-1 in complex with IGFBP-3 and free IGF-1 on the ChP, we used an in vitro model, exposing primary murine ChPE cells to rhIGF-1/rhIGFBP-3 or non-complexed (with IGFBP-3) rhIGF-1. A schematic illustration of the in vitro study outline can be seen in Fig. 4A. The ChPE cells were stimulated with 300 ng rhIGF-1/rhIGPB-3 (corresponding to approx. 60 ng IGF-1, based on a molecular weight ratio of 1:5 rhIGF-1:rhIGFBP-3) or 60 ng rhIGF-1 for 15 min, 2 and 3.5 h, and subjected to Western blot, qRT-PCR, and mass spectrometry (MS) analysis. Western blot analysis resulted in similar activation of the IGF-1R in ChPE cells, visualized by the band intensities of pPKB and pERK1/2, when stimulated with rhIGF-1/rhIGFB-3 or rhIGF-1 for 15 min (Fig. 4B). Full Western blot membranes are provided in Additional File 2: Figure S4. Gene expression analysis, using the RT2 Profiler PCR Array of 84 genes related to angiogenesis (Fig. 4C-I) was performed on ChPE cells exposed to rhIGF-1/rhIGFBP-3, rhIGF-1 or cell culture media only (Control) for 2 h. Exposure to rhIGF-1/rhIGFBP-3 promoted an increase of genes related to immune signaling and cell growth. Further analysis, however, displayed no significant difference except for an upregulation of the brain-specific angiogenesis inhibitor 1 (Bai1) compared to control cells (Vehicle) (Fig. 4E).

**Fig. 5:**
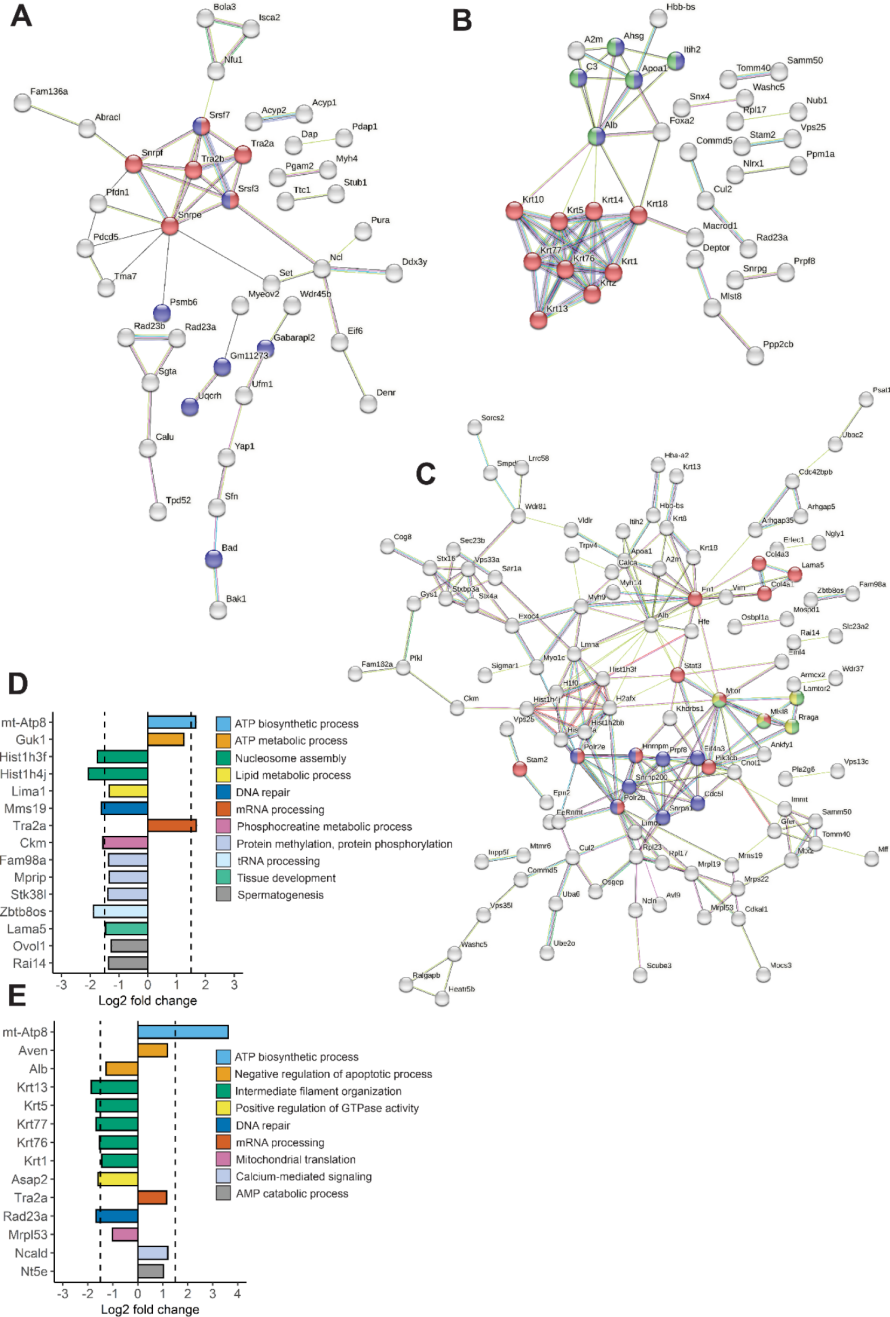
Proteomic analysis of murine ChPE cells after exposure to rhIGF-1/rhIGFBP-3 or rhIGF-1. **A-C.** String network maps of significant changes in protein abundance (confidence level of 95%) in primary murine ChPE cells following exposure to rhIGF-1/rhIGFBP-3 (300 ng/ml) or rhIGF-1 (60 ng/ml), compared to cell culture medium only (Control), for 3.5 h. Colored based on KEGG and Reactome pathway enrichment. Unconnected nodes were removed from network maps. **A.** Increased proteins after rhIGF-1/rhIGFBP-3 stimulation. Red: spliceosome; blue: amyotrophic lateral sclerosis. **B.** Decreased proteins after rhIGF-1 stimulation. Red: formation of the cornified envelope; blue: post-translational protein phosphorylation; green: regulation of IGF transport and uptake by IGFBPs. **C.** Decreased proteins after rhIGF-1/rhIGFBP-3 stimulation. Red: signaling by receptor tyrosine kinases; blue: mRNA splicing - major pathway; green: mTORC1-mediated signaling; yellow: energy dependent regulation of mTOR by LKB1-AMPK. **D-E.** Significant changes in protein abundance when applying stricter significant criteria (fold-change ≥ 1.5 and ≤ -1.5, and Benjamini–Hochberg-corrected p-value ≤ 0.05, dotted lines), after rhIGF-1/rhIGFBP-3 (**D**) or rhIGF-1 (**E**) stimulation. GO terms based on David analysis for individual proteins are displayed

Next, to determine the protein levels upon activation of the IGF-1R in ChPE cells after stimulation with rhIGF-1/rhIGFB-3 or rhIGF-,1 we used MS analysis. ChPE cell were stimulated with rhIGF-1/rhIGFB-3, rhIGF-1 or cell culture media only (Control) for 3.5 h and MS analysis were conducted. Significantly altered protein abundancies in rhIGF-1/rhIGBP-3 and rhIGF-1-treated cells compared to controls (confidence level of 95%, Additional File 4: Table S1) was subjected to String pathway analysis. Inspecting the KEGG and Reactome pathway enrichment, we found increased proteins in spliceosome (Fig. 5A, red nodes), and amyotrophic lateral sclerosis (Fig. 5A, blue nodes) upon rhIGF-1/rhIGFBP-3. RhIGF-1 did not increase sufficient proteins in any pathways in the KEGG or Reactome databases for the String pathway analysis (data not shown). Decreased protein abundancies by rhIGF-1 were found in the pathways of formation of the cornified envelope (Fig. 5B, red nodes), post-translational protein phosphorylation (Fig. 5B, blue nodes), regulation of IGF transport and uptake by IGFBPs (Fig. 5B, green nodes). Decreased protein abundancies by rhIGF-1/rhIGFBP-3 were found in the pathways of signaling by receptor tyrosine kinases (Fig. 5C, red nodes), mRNA splicing - major pathway (Fig. 5C, blue nodes), mTORC1-mediated signaling (Fig. 5C, green nodes) and energy dependent regulation of mTOR by LKB1-AMPK (Fig. 5C, yellow nodes). When applying a stricter significance criterion (fold-change ≥ 1.5 and ≤ -1.5, and Benjamini–Hochberg-corrected p-value ≤ 0.05), few proteins displayed significantly changed abundancies in rhIGF-1/rhIGFBP-3 and rhIGF-1 treated ChPE cells compared to controls. An increase was observed for mitochondrially encoded ATP synthase membrane subunit 8 (mt-Atp8), a subunit in ATP-synthase, in both rhIGF-1 and rhIGF-1/rh/IGBP-3 stimulated ChPE cells, and transformer-2 protein homolog alpha (Tra2a), involved in mRNA processing, in the rhIGF-1/rhIGFBP-1 stimulated cells (Fig. 5D and E). A decrease was observed for proteins involved in filament organization and nucleosome assembly in both groups compared to the control (Fig. 5D and E). Complete protein names, gene ontology, KEGG and Reactome pathway enrichments terms can be seen in Additional File 4: Table S2, Additional File 5: Table S3, and Additional File 6: Table S4.

## Discussion

In this work, we show that s.c. administrated rhIGF-1/rhIGFBP-3 reaches the ChP in the lateral and third ventricles as well as the perivascular and subarachnoid space in the preterm rabbit pup. In addition, we show that the timing of s.c. administration of rhIGF-1/rhIGFBP-3 affects the uptake of IGF-1 to the circulation which in turn relates to higher concentrations in the CSF. Additionally, administered rhIGF-1/rhIGFBP-3 displayed a time-dependent activation of the IGF-1R in the ChP of the immature brain in the preterm rabbit pup. Other key findings are: (i) expression of genes paramount for the development of the ChP exhibits a clear relation to postnatal age, and (ii) rhIGF-1/rhIGFBP-3 and rhIGF-1 exhibit similar effect on ChPE cells in vitro.

The ChP has traditionally been regarded as a simple epithelial sheet of cells, tightly bound together by tight junction proteins, whose main function is to filter the circulatory plasma to produce CSF [26, 27]. However, the ChP has been shown to be constituted of a plurality of cells in the stroma including, mesenchymal-, glial-, immune-, neuronal- and fenestrated endothelial cells [28], giving rise to the potential for the ChP to be involved in a number of processes, and to produce a span of molecules affecting the brain parenchyma [29]. The development of the ChP has been increasingly investigated showing the importance of ChP, especially in the immature brain of the preterm subject [26, 27, 29–32].

It has been indicated that the interaction of blood borne molecules with the ChP may be temporally dependent, at least concerning IGF-1 and IGF-1/IGFBP-3 [33]. For instance, Bunn et al., showed that children < 6 months of age had significantly higher CSF levels of IGF-1, IGFBP-1 and IGFBP-3 than children older than 6 months [33]. The data presented here displays that systemic rhIGF-1/rhIGFBP-3 has an opportunistic window immediately after preterm birth to affect the immature ChP and reach the CSF in the preterm rabbit pup. We observed that the systemic serum concentration of IGF-1 is the main determinant of the transfer into the CSF, however, only pups receiving rhIGF-1/rhIGFBP-3 at a postnatal age of 0 h display an uptake of IGF-1 to the CSF that correlates with the uptake to the serum.

The uptake of IGF-1 from the s.c. space to the blood at 0 h postnatal age could be caused by low renal filtration of the IGF-1/IGFBP-3 complex in the preterm rabbit pups administered early after birth [34]. An additional factor is hydration. As pups administered at 0 h postnatal age have not been fed, the circulatory volume might be reduced compared to older pups, thus potentially leading to an increase in the serum IGF-1 concentration. Furthermore, we observed that the relative transfer from circulation to the CSF, i.e. CSF/serum IGF-1 ratio, is fairly constant over the first 3-days studied here. Moreover, the uptake of rhIGF-1/rhIGFBP-3 is correlated with the activation of the IGF-1R, as displayed by increased pPKB and pERK1/2 following rhIGF-1/rhIGFBP-3 treatment at 0 h. Therefore, it could be speculated that the higher absolute concentration of IGF-1 in the CSF is dependent on both the binding to the IGF-1R in the ChP, and a putative increased serum concentration driven diffusion rate or transcellular translocation in the blood-CSF barrier in 0 h treated pups [35, 36]. The translocation of IGF-1 through the ChP to the CSF has indeed been described to involve the IGF-1R [9]. Taken together, this indicates that the possibility for systemic IGF-1 to affect the immature brain is primarily serum concentration driven.

Despite years of investigating the presence of IGF-1 in the CNS, following systemic administration [2, 9, 37–39], to the best of our knowledge, there is to this date no holistic imaging of IGF-1 in the preterm brain available. Therefore, to establish a comprehensive view of the uptake of systemic IGF-1 to the CNS we used light-sheet imaging in combination with confocal- and transmission electron microscopy, and chromogenic IHC to evaluate the distribution of IGF-1 in the immature brain of the preterm rabbit pup following administration of rhIGF-1/rhIGFBP-3. We found a substantial presence of rhIGF-1/rhIGFBP-3 in the ChP most pronounced in the third and lateral ventricles, the SFO, as well as the perivascular- and the subarachnoid space. Our findings, based on confocal microscopy, primarily indicate an accumulation of rhIGF-1/rhIGFBP-3 at the endothelial barrier of both the ChP and the SFO. Using high resolution imaging made possible by TEM, we observed that the rhIGF-1/rhIGFBP-3 complex appears to translocate through the ChP. Interestingly, the administration of non-labeled rhIGF-1/rhIGFBP-3 caused an increased IGF-1 immunoreactivity (shown by anti-IGF-1 IF and chromogenic IHC, Additional file 2, Fig. S1) in the hippocampus and cerebellum, developmentally important structures found adjacent to the ventricles. Although we were not able to fully elucidate if the increased immunoreactivity is caused by an increased endogenous production of IGF-1, or an intrusion of systemic IGF-1, the absence of major immunoreactivity observed following administration of biotin-, FITC- and AF647-labeled rhIGF-1/rhIGFBP-3 indicate that it is endogenous IGF-1.

The ChP of the preterm rabbit pup born at embryonic day 29, corresponding to an approximate human gestational age of 24–28 weeks, is presumed not to be fully developed [10, 21, 29]. Our data shows that the expression of genes is linked to the development of the brain and the ChP, i.e. TTR [22], OTX2 [23], BMP4 [24, 25] and FOLR1 [40, 41], increased significantly in the ChP of preterm rabbit pups at 77 h postnatal age compared to 5 and 29 h postnatal age. Administered rhIGF-1/rhIGFBP-3 was not observed to have a significant impact on the genes and timeframe evaluated here. In the study by Richardson et al., the mRNA level of TTR was observed to increase by 40-fold from E12.5 until birth reaching a peak at two days before birth, just prior to the highest brain development rate in the rat [22]. Thus, the TTR levels in the 77 h postnatally aged rabbit pups possibly reflects the establishment of the ChP, and that the corresponding need for IGF-1 in the immature brain is reduced. In fact, this correlates with reduced IGF-1R density in the brain of the preterm rabbit pup at 96 h postnatal age compared to 4 h postnatal age [3]. In our study, the peak OTX2 expression was found at 77 h postnatal age (corresponding to 0 days postnatal age of term pups). This corresponds to a delayed expression compared to embryonic mice [23], but could possibly reflect similarity in rabbit ChP development to humans where the expression of ChP OTX2 was observed to increase by postnatal age 7 to 9 [42]. Importantly, we did not evaluate this at a later timepoint, and consequently cannot exclude the possibility of additional changes in expression of OTX2 in the ChP as the rabbit ages.

IGFPBs have been proposed to exhibit effects on brain development and cognition, without the conjugation to IGFs [43, 44]. We therefore compared the effects of rhIGF-1/rhIGFBP-3 or rhIGF-1 alone, on murine in vitro ChPE cells. Both rhIGF-1/rhIGF-1 and rhIGF-1 activated the IGF-1R and increased levels of proteins involved in ATP synthesis and mRNA processing at 15 min and 3.5 h after exposure, respectively. Interestingly, both rhIGF-1/rhIGFBP-3 and rhIGF-1 reduced levels of proteins in pathways in the string pathway analysis concerning IGF-1 and cell growth, including IGF uptake, tyrosine receptor signaling and mTOR signaling. As the IGF-1R activation study was conducted at 15 min exposure of the ChPE cells, and the proteomic analysis at 3.5 h exposure, a time-dependent window for IGF-1 to interact could perchance be present in the current in vitro model. It could therefore be that the window of peak growth is closed earlier in stimulated compared to unstimulated cells. Taken together, no major differences on the impact of ChPE cells by rhIGF-1/rhIGFBP-3 or rhIGF-1 were observed in the current study.

## Conclusions

**Fig. 6:**
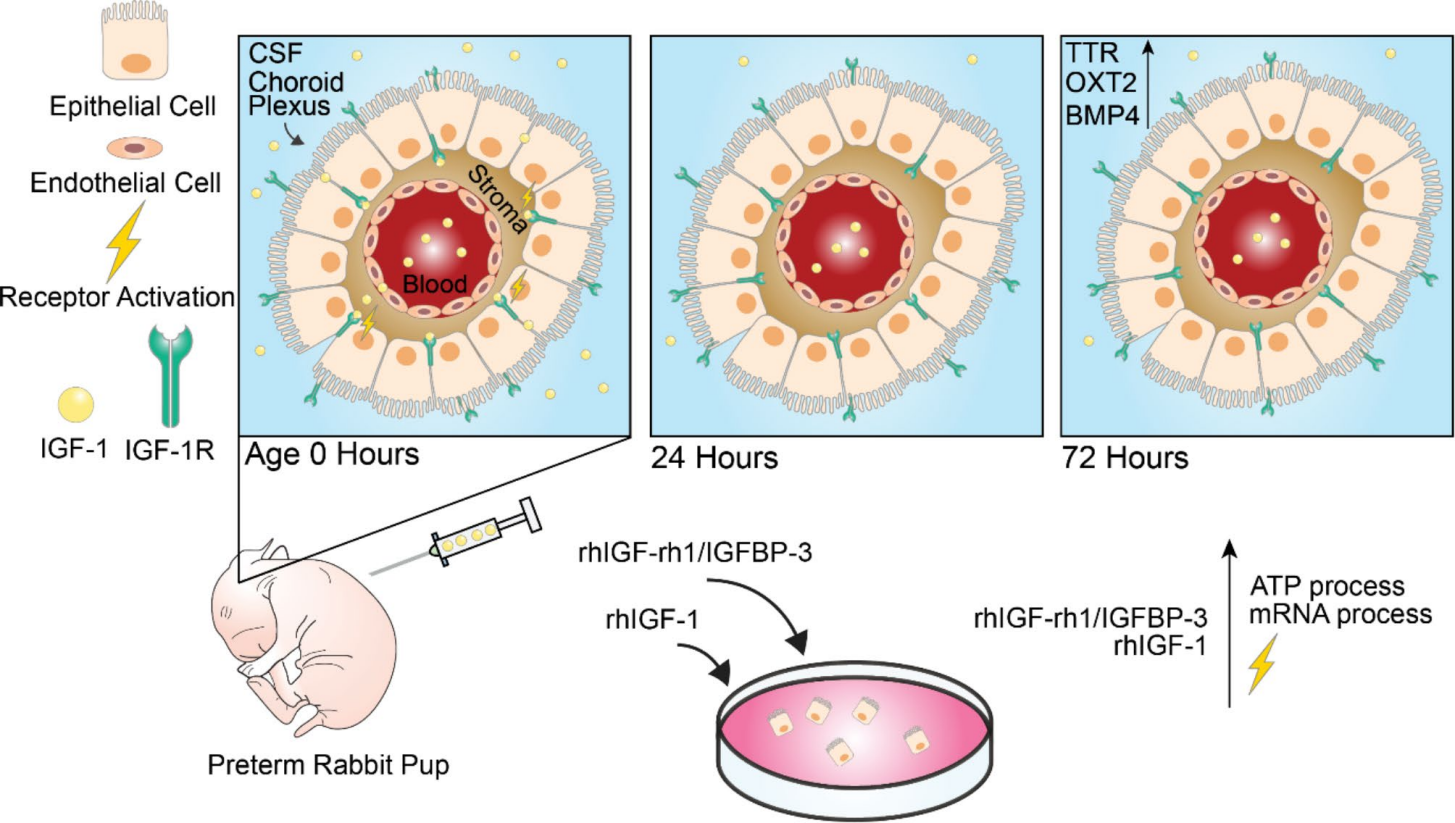
Schematic illustration of the main findings in the present study. Subcutaneous administration of rhIGF-1/rhIGBP-3 to preterm rabbits (E29), results in a higher level of IGF-1 in serum and CSF at postnatal age at administration of 0 h compared to 24 and 72 h. This is in turn correlated with an increased IGF-1R activation in the ChP at a postnatal age of 5 h, and expression of genes involved in the ChP development. Stimulating ChPE cells with rhIGF-1/rhIGFBP-3 or rhIGF-1 results in an activation the IGF-1R and an increase in protein synthesis involved in ATP production and mRNA processing

The data in the present study, depicted in the schematic illustration in Fig. 6, shows that systemic rhIGF-1/rhIGFBP-3 reaches the ventricles and the perivascular- and subarachnoid space and translocates through the ChP in the immature brain of the preterm rabbit pup. We observed that the postnatal age after preterm birth affects the translocation, the IGF-1R activation in the ChP, and the expression of genes paramount for the development of the ChP. By using a primary murine in vitro ChPE cell culture, we show that rhIGF-1/rhIGFBP-3 and free (non IGFBP-3 complexed) rhIGF-1 exhibit a similar effect on the ChP. More research is needed to fully understand the temporal interactions between IGF-1 and other growth factors with the ChP and the immature brain.

### Electronic supplementary material

Below is the link to the electronic supplementary material.

Supplementary Material 1

Supplementary Material 2

Supplementary Material 3

Supplementary Material 4

Supplementary Material 5

Supplementary Material 6

## Data Availability

Data available upon request from the corresponding author.

## Abbreviations

ChP: Choroid plexus
ChPE: Choroid plexus epithelia
IGF-1: Insulin-like growth factor 1
IGF-1R: IGF-1 receptor
RhIGF-1/rhIGFBP-3: recombinant human IGF-1/IGF binding protein 3
SFO: Subfornical organ
IVH: Intraventricular hemorrhage
FITC: Fluorescein isothiocyanate
TEM: Transmission electron microscopy
TTR: Transthyretin
OTX2: Orthodenticle homeobox 2
BMP4: Bone morphogenetic protein 4
BMP7: Bone morphogenetic protein 7
AQP1: Aquaporin 1
AQP5: Aquaporin 5
ALB: Albumin
FOLR1: Folate receptor 1
TGFβ3: Transforming growth factor beta
HPRT: Hypoxanthine phosphoribosyltransferase 1
Bai-1: Brain-specific angiogenesis inhibitor 1
Nos3: Nitric oxide synthase 3
Plg: Plasminogen
Ccl11: C-C motif chemokine 11
Cxcl2: Chemokine (C-X-C motif) ligand 2
Egf: Epidermal growth factor
mt-Atp8: ATP synthase membrane subunit 8
Tra2a: Transformer-2 protein homolog alpha
CNS: Central nervous system
CSF: Cerebrospinal fluid
AF: Alexa Flour
PFA: Paraformaldehyde
TX: Triton X-100
DAPI: 4′,6-diamidino-2-phenylindole
DCM: Dichloromethane
EtCinn: Ethyl cinnamate
DTT: Dithiothreitol
QRT-PCR: Quantitative real-time PCR
SDS-PAGE: Sodium dodecyl-sulfate polyacrylamide gel electrophoresis
IHC: Immunohistochemistry
IF: Immunofluorescence
MS: Mass spectrometry
BSA: Bovine serum albumin
GO: Gene Ontology
KEGG: Kyoto Encyclopedia of Genes and Genomes
CD31: Cluster of differentiation 31
