# Supplementary material for: Characterization of choroid plexus in the preterm rabbit pup following subcutaneous administration of recombinant human IGF-1/IGFBP-3
Source: Fluids Barriers CNS. 2023 Aug 15;20:59. doi: 10.1186/s12987-023-00460-1 (PMC10426218; doi:10.1186/s12987-023-00460-1)
Supplement: Supplementary file 2 — Supplementary Material 2 [file 12987_2023_460_MOESM2_ESM.docx]

**Additional file 2**


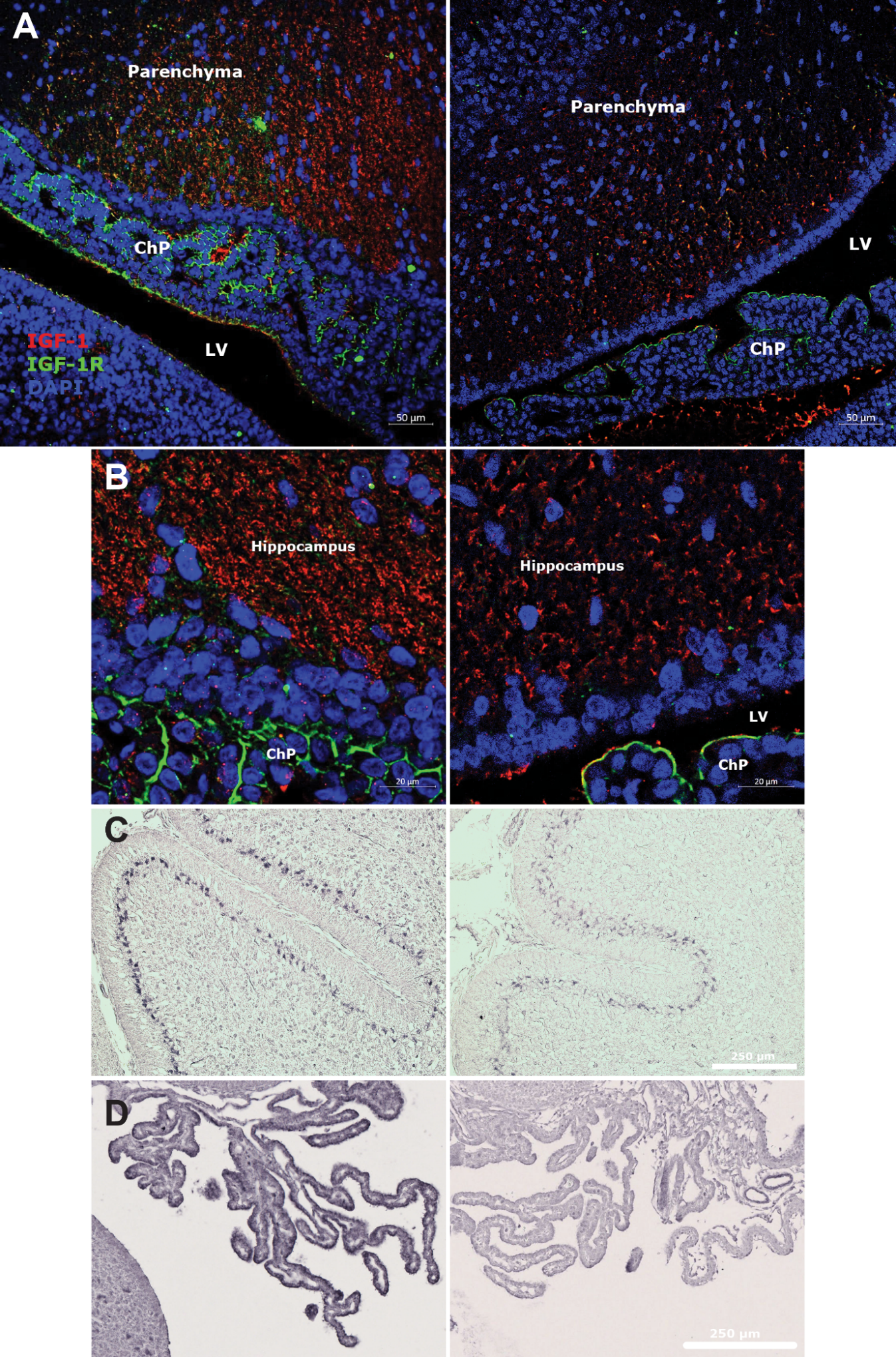


**Supplementary Figure 1. Increased IGF-1 immunoreactivity in the preterm brain upon s.c.** **rhIGF-1/rhIGFBP-3 administration. A-D** Representative confocal and histochemistry microscopy images displaying IGF-1 immunoreactivity in the parenchyma upon systemic rhIGF-1/rhIGFBP-3 administration (red, A, left) and hippocampus (red, B, left) compared to vehicle treated (red, A, right and red, B, right). A and B also displays an immunoreactivity of IGF-1R (green) in the ChP in rhIGF-1/rhIGFBP-3 treated animals. Cell nuclei were counterstained with DAPI. IGF-1 immunoreactivity in the cerebellum (blue, C, left) and ChP in the fourth ventricle (blue, D, left) upon systemic rhIGF-1/rhIGFBP-3 administration compared to vehicle (blue, C and D, left).


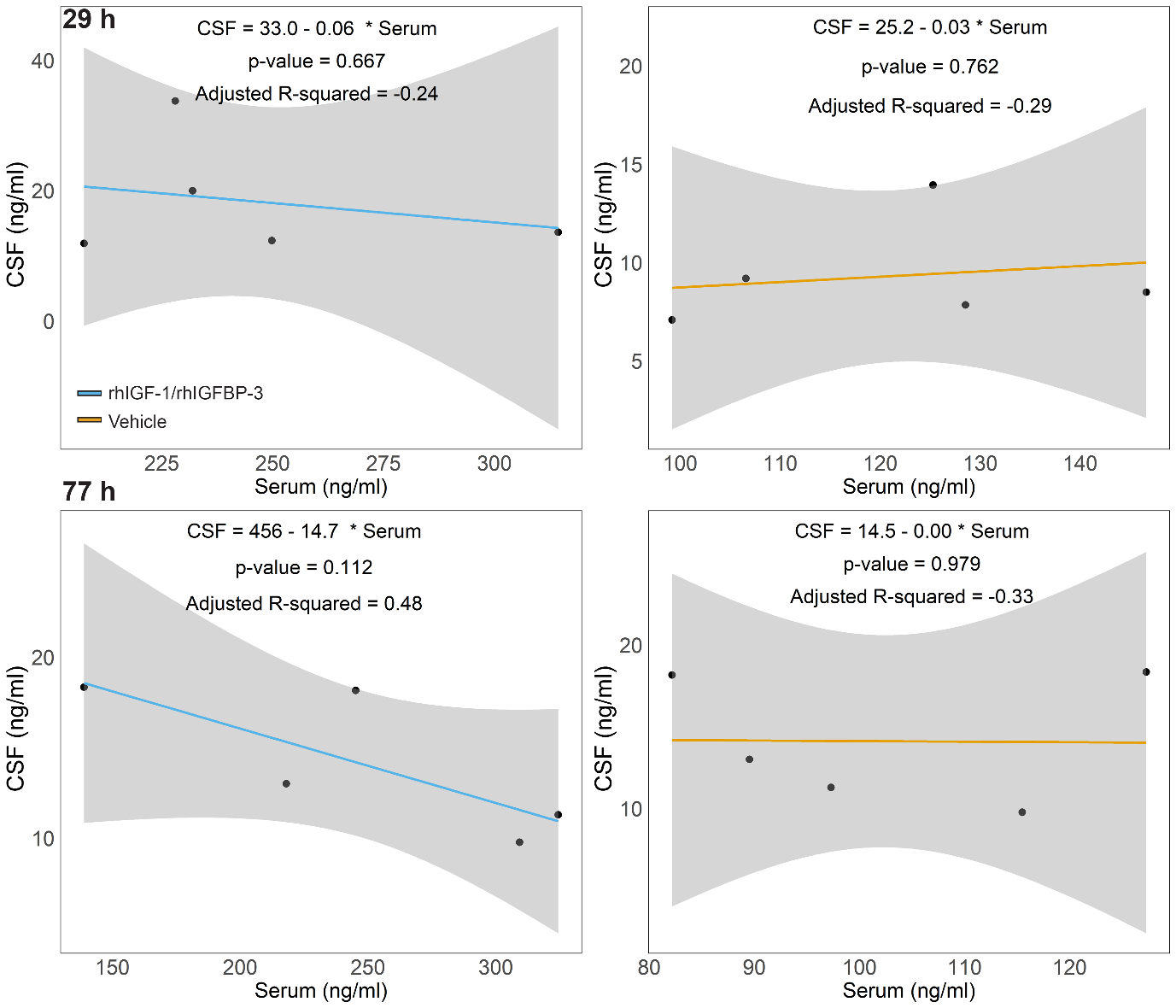


**Supplementary Figure 2. Correlation between IGF-1 levels in serum and CSF.** Linear model displaying the relation between the IGF-1 levels in serum and CSF in 24 (29) hours (top panel) and 72 (77) hours (lower panel) postnatal age pups, corresponding time-point for termination within parentheses. Dark grey area represents the 95% confidence level.


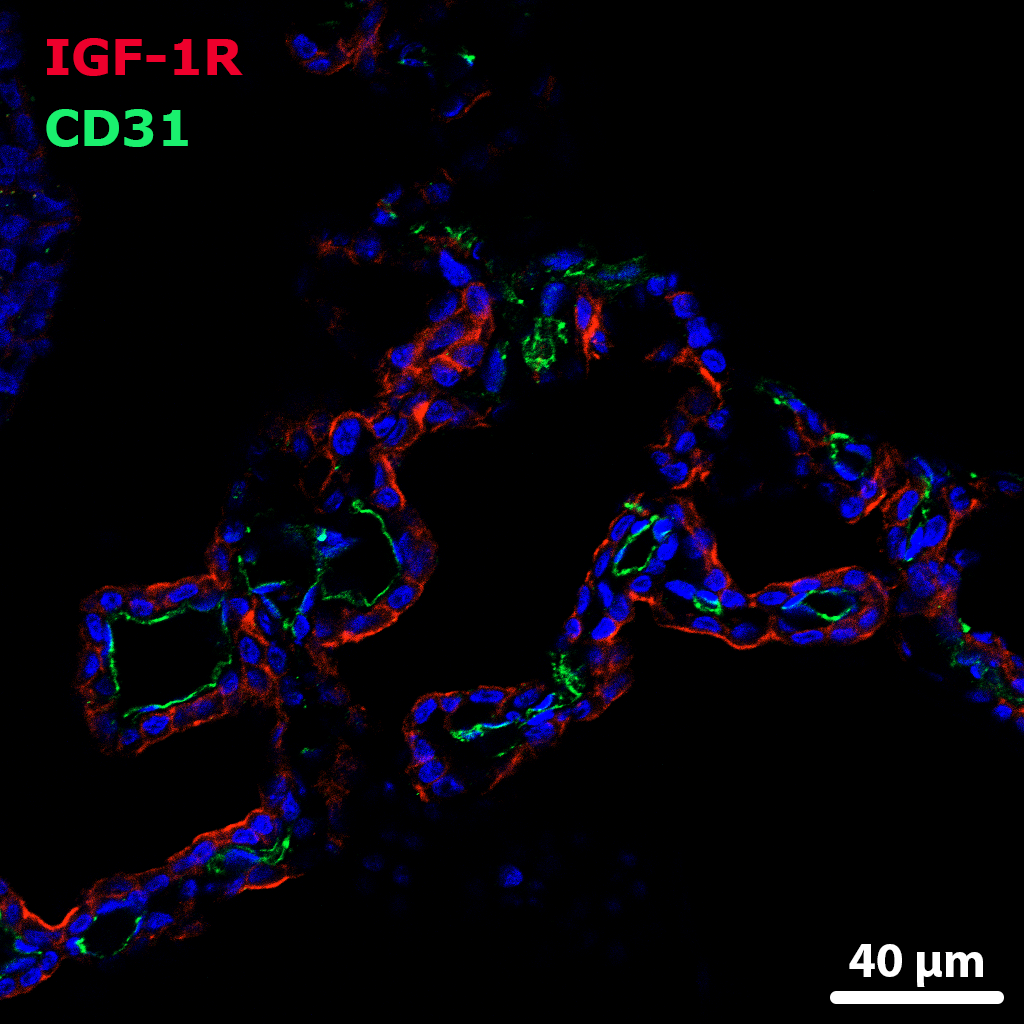


## **Supplementary Figure 3. Location of IGF-1 receptor at the ChP.** Representative confocal images if IGF-1R (red) and CD31 (green) in the ChP in preterm rabbit pup brain. Data was collected from 2 independent experiments.

##
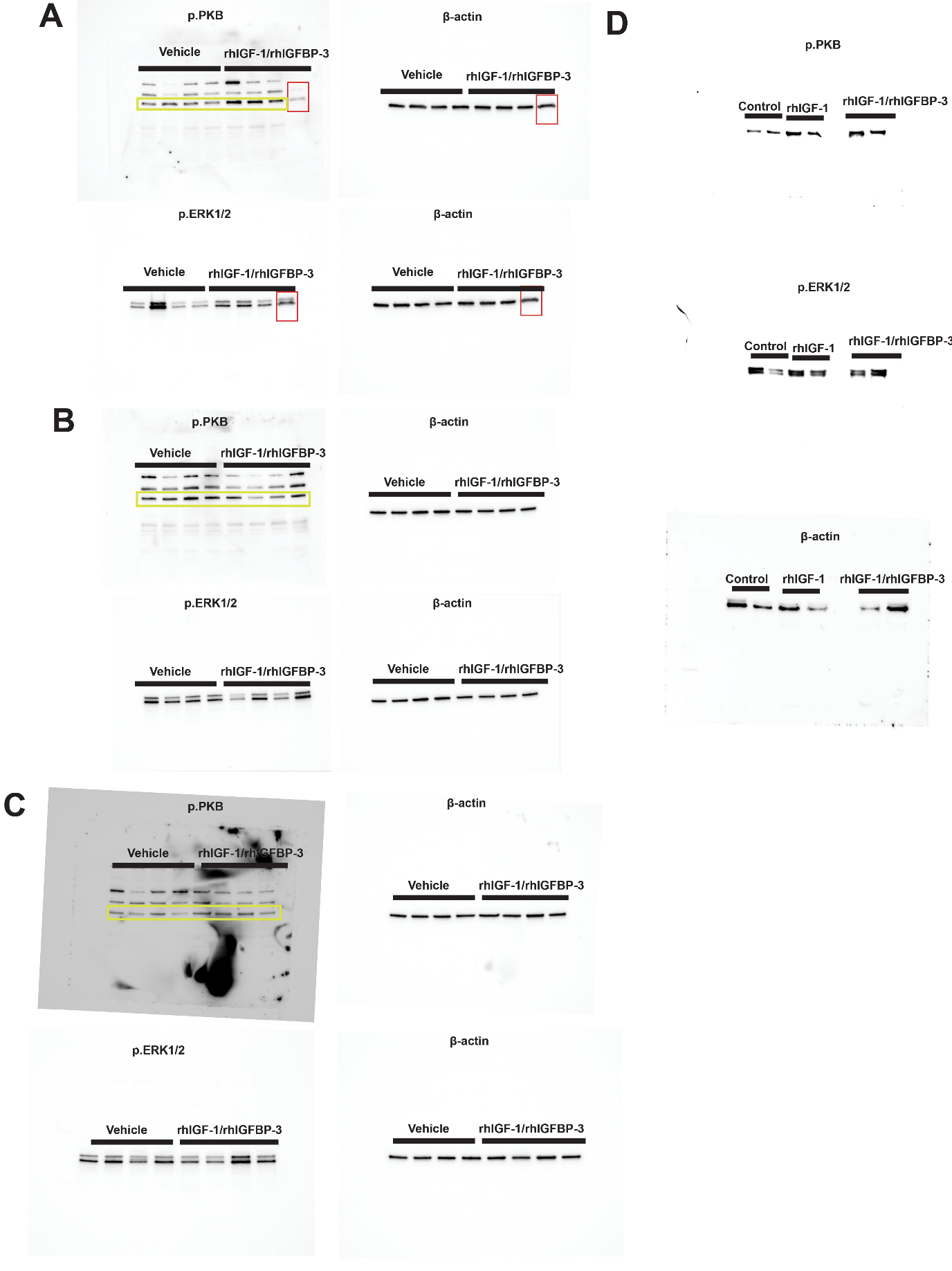


**Supplementary Figure 4. Western blot membranes used in the present study. A -C** Western blot analysis of p.PKB and p.ERK1/2 (in ChP from preterm rabbit pups 5 hours after s.c. administration of rhIGF-1/rhIGFBP-3 (8 mg/kg), administered at 0 (**A**), 24 (**B**) or 72 hours (**C**) postnatal age. Red square indicates excluded bands from the study due to technical difficulties. Yellow square indicate band of approx. 60 kDa, corresponding to p.PKB (Thermo Fisher, 44-621G), used in the analysis presented in Figure 2E. **D** Western blot analysis of p.ERK1/2 and p.PKB in primary murine ChPE cells following exposure to rhIGF-1/rhIGFBP-3 (300 ng/ml) or rhIGF-1 (60 ng/ml) for 15 minutes.
